# *Salmonella* adhesion is decreased by hypoxia due to adhesion and motility structure crosstalk

**DOI:** 10.1186/s13567-023-01233-2

**Published:** 2023-10-24

**Authors:** Krzysztof Grzymajło, Agata Dutkiewicz, Joanna Czajkowska, Ewa Carolak, Adrianna Aleksandrowicz, Wiktoria Waszczuk

**Affiliations:** Present Address: Faculty of Veterinary Medicine, Department of Biochemistry and Molecular Biology, Wrocław University of Environmental and Life Sciences, Wrocław, Poland

**Keywords:** *Salmonella*, type 1 fimbriae, adhesion, motility, hypoxia, infection, microbiome, flagella

## Abstract

Initial stages of *Salmonella* Typhimurium infection involve a series of coordinated events aimed at reaching, attaching to, and invading host cells. Virulence factors such as flagella, fimbriae, and secretion systems play crucial roles in these events and are regulated in response to the host environment. The first point of contact between the pathogen and host is the intestinal epithelial layer, which normally serves as a barrier against invading pathogens, but can also be an entry site for pathogens. The integrity of this barrier can be modulated by the hypoxic environment of the intestines, created by the presence of trillions of microbes. Variable oxygen concentrations can strongly affect many functions of the gut, including secretion of cytokines and growth factors from the host site and affect the ability of *Salmonella* to persist, invade, and replicate. In this study, we investigated the first stages of *Salmonella* Typhimurium infection under hypoxic conditions in vitro and found that low oxygen levels significantly decreased bacterial adhesion. Using adhesion and motility assays, biofilm formation tests, as well as gene expression and cytokine secretion analysis, we identified a hypoxia-specific cross-talk between the expression of type 1 fimbriae and flagella, suggesting that altered flagellin expression levels affect the motility of bacteria and further impact their adhesion level, biofilm formation ability, and innate immune response. Overall, understanding how *Salmonella* interacts with its variable host environment provides insights into the virulence mechanisms of the bacterium and information regarding strategies for preventing or treating infections. Further research is required to fully understand the complex interplay between *Salmonella* and its host environment.

**Supplementary Information:**

The online version contains supplementary material available at 10.1186/s13567-023-01233-2.

## Introduction

Following oral infection and survival in the acidic pH of the stomach, *Salmonella* has a good chance of colonizing the intestines. The initial stages of *Salmonella* infection are determined by a series of coordinated events that focus on reaching, attaching to, and invading host cells. Expression of crucial virulence factors responsible for these events, such as flagella, fimbriae, and secretion systems, in the context of the host environment determines the potential success of the infection. *Salmonella* uses flagella to move along the host tissue surface before finding an appropriate binding site, fimbriae to attach to the cell surface, and invasion systems to actively enter host cells [1–3]. The expression of virulence factors comes at a cost, and there is an obvious trade-off between the benefits of virulence and fitness costs associated with maintaining these traits. However, specific interplay among these virulence factors during *Salmonella* infection is not well understood.

Flagella, long helical filaments attached to rotary motors, enable bacteria to swim in liquids and swarm over the surface [4]. They have also been implicated in the invasion process of *Salmonella*, mostly by enhancing bacterial motility and enabling it to reach the invasion site [5]. It has also been demonstrated that methylated flagella facilitate the adhesion of *Salmonella* to epithelial cells [6]. Among the adhesive structures of *Salmonella*, type 1 fimbriae (T1F) are one of the most extensively studied [3]. It has been previously shown that T1F play an important role in adhesion to host cells and animal infection. Moreover, variations in the T1F phenotype are associated with variable binding abilities of *Salmonella* and its host specificity [7–10]. Both the flagella and T1F have been shown to induce proinflammatory cytokine expression [11, 12] and play a role in biofilm formation [13]. Finally, the key player in *Salmonella* invasion is the type 3 secretion system (T3SS), encoded by the *Salmonella* Pathogenicity Island 1 (SPI-1) [1, 14]. This system is directly responsible for invasion via injection of *Salmonella* proteins into host cells, rearrangement of the actin skeleton, and induction of inflammatory diarrhea [15]. All these systems undergo extensive crosstalk during *Salmonella* infection, and their expression is tightly regulated depending on environmental circumstances.

The initial stages of *Salmonella* invasion are affected by a hostile host environment and trillions of microorganisms (microbiota) inhabiting the intestines. Most of the microbial species present in the microbiota are anaerobes, which decrease local oxygen levels and create a highly hypoxic environment within the intestines [16]. This so-called physiological hypoxia strongly affects many functions of the gut, such as secretion of cytokines and growth factors [17, 18], and increases barrier integrity in a hypoxia-inducible factor 1 (HIF-1)–dependent manner [19]. Moreover, regulation of oxygen levels by the inhabiting microbes may provide additional protection against invading pathogens [20]. However, *Salmonella*, as a facultative anaerobe, can benefit from both normoxic as well as hypoxic conditions. Hypoxia promotes replication of *Salmonella* in macrophages; however, its effect on the initial stages of *Salmonella* infection in the intestines is still unclear [21]. Inflamed and infected tissues of the gut display low oxygen concentration, which affects not only the composition of the gut microbiota and host cell reactions, but also the expression of virulence factors and host–pathogen interactions [20, 22]. On the contrary, *Salmonella* can exploit its aerobic respiration ability to efficiently compete with the microbiota [20].

The intestinal epithelial layer creates a barrier against invading pathogens and produces inflammatory cytokines, chemokines, and antimicrobial peptides. Simultaneously, it offers an entry site for many pathogenic bacteria. Therefore, intestinal epithelial cell (IEC) lines, especially non-transformed and non-tumor–derived cells, with significant morphological and functional similarities to normal enterocytes, may serve as an appropriate model for investigating various environmental factors impacting host–pathogen interactions, such as low oxygen tension/hypoxia [23]. It is widely known that the cellular response to low oxygen levels involves stabilization of HIF-1, which induces the expression of several genes that promote angiogenesis, glucose metabolism, and apoptosis, all of which contribute to the restoration of oxygen homeostasis in systemic response to hypoxic stress [24]. Interestingly, HIF-1 was found to increases the integrity of the epithelial cell barrier in two major ways: first, by supporting IEC proliferation, and second, by securing stabilization of the tight junction barrier [25]. Additionally, HIF-1 is able to regulate the host’s innate immune response to invading pathogens [26].

The purpose of this study was to elucidate the early stages of *Salmonella* Typhimurium infection in intestinal cells under hypoxic conditions, particularly focusing on the role of T1F-dependent adhesion. By conducting an in-depth analysis of bacterial adhesion, motility, biofilm formation, and the host's innate immune response in a low-oxygen environment, we aimed to uncover new insights into the mechanisms of *Salmonella* infection and host adaptation. Here, we demonstrate that a low oxygen environment significantly decreased *Salmonella* Typhimurium adhesion level. We associated the altered adhesion phenotype with reduced motility of the pathogen and a significantly decreased ability to form biofilms due to decreased flagellin expression. Finally, we found that hypoxia significantly reduced IL-8 expression and secretion during *Salmonella* Typhimurium infection compared to strongly overexpressed cytokines at normal oxygen levels. This could contribute to our understanding of the diverse infection strategies used by different pathogens and may help in developing more effective interventions for *Salmonella* and other similar infections.

## Materials and methods

### Bacterial strains and culture conditions

Bacterial strains and plasmids used in this study are listed in Tables 1 and 2, respectively. All the strains were grown routinely in Luria–Bertani (LB) medium at 37 °C without agitation. The strains were cultured in conditions of normoxia (5% CO_2_) and hypoxia (1% O_2_, 5% CO_2_, 94% N_2_). In all experiments, strains after the third passage were used [27].

**Table 1:** **Bacterial strains used in this study.**

Strains	Strain tag	Characteristic	References
*Salmonella* Typhimurium	*S*TmWT	*Salmonella* Typhimurium SL1344	Monack lab strain collection [55]
*Salmonella* Typhimurium SL1344 ∆*fimH*	*S*Tm∆*fimH*	*Salmonella* Typhimurium SL 1344 with *fimH gene* knockout	Department of Biochemistry and Molecular Biology [27]
*Escherichia coli* DH5α λpir		*Escherichia coli* DH5α with integrated conjugal transfer functions	Monack lab strain collection [55]
*Escherichia coli* S17-1 *λpir*		*Escherichia coli* S17-1 with integrated conjugal transfer functions	Monack lab strain collection [55]

**Table 2:** **Bacterial plasmids used in this study.**

Plasmid	Characteristic	References
pEMG	Suicide plasmid; KmR	[56]
pSW-2	Plasmid for m-toluate-inducible expression of the I-SceI enzyme; GmR	[56]
pEMG ∆*fimH*	Suicide plasmid; KmR; with ∆*fimH* insert	[27]

### Cell lines and cultures

Porcine intestinal epithelial cell line IPEC-J2 was cultured in DMEM (Biowest, Nuaillé, France) supplemented with 10% fetal bovine serum (FBS; Biowest), 2 mM glutamine, and antibiotics (penicillin, streptomycin; Biowest). For adhesion assays, cells were seeded in 24-well plates (Greiner Bio-One, Frickenhausen, Germany) and grown under normoxic (5% CO_2_) or hypoxic (1% O_2_, 5% CO_2_, 94% N_2_) conditions until a confluent monolayer was obtained.

### Determination of the growth curves

Growth curves were obtained for all the *Salmonella* isolate cultures, both in normoxic and hypoxic conditions. OD_600_ of the overnight cultures was measured and adjusted to 0.05. Bacterial cultures were grown until they reached OD_600_ = 0.5. Next, 1 mL of each culture was centrifuged and washed twice with 0.9% NaCl. Later, OD_600_ was measured, and volumes of the cultures were adjusted to obtain a bacterial density of 5 × 10^6^ CFU/mL. The bacteria were then transferred into 96-well plates, 200 µL per well, in triplicates. OD_600_ in normoxic conditions was measured using a SPARK (TECAN, Mannedorf, Switzerland) plate reader for 12 h at 37 °C, at 15-min intervals preceded by 30 s of mixing. Meanwhile, to obtain hypoxic conditions, the cultures were incubated in an incubator with an appropriate gas supply (1% O_2_, 5% CO_2_, 94%N_2_) at 37 °C. OD_600_ was measured hourly for 12 h using a SmartSpec Plus spectrophotometer (Bio-Rad, Hercules, USA).

### Yeast agglutination assay—type 1 Fimbriae expression measurement

Bacteria were grown as described above, collected by centrifugation, washed with PBS, and adjusted to OD_600_ = 1. Two-fold serial dilutions in PBS were prepared and added to 96-well microplates. An equal volume (100 μL) of 0.5% *Saccharomyces cerevisiae* (0.5 mg/100 μL) was added to each well and gently mixed*.* The plates were incubated at 37 °C for 30 min in normoxia. The highest dilution of the suspensions that showed agglutination was defined as the titer endpoint.

### Adhesion assay

Confluent monolayers of IPEC-J2 cells in a 24-well plate were washed once with PBS. The medium was replaced with DMEM without added fetal bovine serum (FBS) or antibiotics. Before performing the assay, the bacteria were washed once with PBS and diluted to an optical density corresponding to 2 × 10^8^ CFU/mL (Multiplicity of infection; MOI = 100) or 2 × 10^7^ CFU/mL (MOI = 10) and allowed to interact with the IPEC-J2 cells for 2 h at 37 °C in 5% CO_2_ (normoxic conditions) or 37 °C in 1% O_2_, 5% CO_2_, 94% N_2_ (hypoxic conditions). If indicated, the bacterial cells were centrifuged for 5 min at 250 × *g* at 25 °C before the infection period. After 2 h of incubation, the medium was discarded and cells were washed three times with PBS and lysed for 10 min with PBS containing 0.1% Triton X-114 (Sigma Aldrich, Saint Louis, Missouri, USA). The number of CFU in each well was determined by plating serial dilutions of cell lysates on LB plates. At least five independent experiments, with three technical replicates for each *Salmonella* strain, were performed under each condition. To verify the differences in bacterial growth during the experiments conducted under various growth conditions (normoxia vs. hypoxia), two sets of plates containing only *Salmonella* isolates were incubated for 2 h under both growth conditions, and then serially diluted and plated for CFU calculation.

### Motility on soft agar

Two μL of *Salmonella* cultures (6 × 10^5^ CFU per 2 μL) after the third passage grown in LB broth under T1F–inducing conditions were placed on 0.3% agar LB plates, and the plates were incubated for 6 h at 37 °C in normoxia and hypoxia. Diameters of the diffusion zones were measured after 3, 4, 5, and 6 h of incubation. At least three independent experiments with three replicates for each strain were performed.

### Assessment of biofilm biomass

*Salmonella* isolates after the third passage were used in the experiment. OD_600_ of each isolate was adjusted to 1 and diluted to 3 × 10^8^ CFU/mL in LB. Next, 2 mL of the bacterial cultures were transferred into 24-well plates (Greiner Bio-One, Frickenhausen, Germany) and incubated at 37 °C for 24, 48, and 72 h under normoxic and hypoxic conditions. After incubation, the medium was carefully discarded, and the biofilm formed on the bottom of the wells was dried for 10 min at 25 °C. Later, 1 mL of 0.1% crystal violet in water was added to all the wells, and the plate was incubated for 10 min at 25 °C. The solutions were then discarded, and the wells were rinsed twice with water. The bound crystal violet was dissolved in 1 mL of 96% ethanol and shaken for 10 min, and three technical repetitions of 100 μL of the solution from each well was transferred to a new 96-well plate. Absorbance was measured at 570 nm using a Spark plate reader (Tecan, Zürich, Switzerland). The experiment was repeated twice in triplicate.

### ELISA

IPEC-J2 cells were infected with *Salmonella* isolates according to the adhesion assay protocol described above. After incubation for 2 h, supernatants were collected from each well. Untreated cells were used as controls. Each supernatant was centrifuged for 5 min at 2500 × *g* at RT to eliminate bacteria and other debris. ELISA was performed using the Porcine IL-8 ELISA Kit (Invitrogen, Austria) according to the manufacturer’s instructions. Absorbance was measured at 450 nm using the SPARK plate reader (Tecan, Zürich, Switzerland).

### Total RNA isolation and qPCR

Total RNA was extracted from *Salmonella* isolates after the third passage using the RNeasy Mini Kit (Qiagen, Hilden, Germany). Isolation was performed by adding RNAprotect Bacteria Reagent (Qiagen) to stabilize RNA in the bacteria, allowing accurate analysis of gene expression, according to the manufacturer’s protocol with minor modifications.

Total RNA was extracted from IPEC-J2 cells after incubation for 2 h with *Salmonella* isolates according to the adhesion assay protocol mentioned above. Cells not treated with *Salmonella* were used as controls. After incubation, isolation was performed using the RNeasy Mini Kit (Qiagen) according to the manufacturer’s protocol. RNA quantity and quality were assessed using Nanodrop 2000c (Thermo Fisher Scientific, Waltham, MA, USA).

Total RNA was reverse transcribed using the High Capacity cDNA Reverse Transcription Kit (Thermo Fisher Scientific) according to the manufacturer’s instructions. Relative amounts of mRNA were quantified by qPCR using a CFX thermocycler (Bio-Rad). cDNA thus obtained was amplified using the following conditions: 5 min incubation at 95 °C for initial denaturation, followed by 35 cycles of 20 s denaturation at 95 °C, 20 s annealing at 56 °C, and 20 s elongation at 72 °C. The melt curve analysis was performed from 65 to 95 °C with an increment of 0.5 °C for 5 s. Expression of the target genes was determined using the EvaGreen dye (Biotinum, Fremont, CA, USA) and normalized to 16S RNA (stable under normoxia and hypoxia [Additional file 7]) or actin as a reference.

Relative quantification of gene expression was performed using the comparative Ct method, using *S*TmWT grown under normoxic conditions as an untreated control/calibrator sample. Biological (minimum three replicates) and technical (minimum two replicates) replicates were used to derive the final data. The primer sequences used are listed in Additional file 1.

### RNA-sequencing

*Salmonella* strains (*S*TmWT and *S*TmΔ*fimH*) were grown in T1F-inducing conditions under hypoxia and normoxia as described above. Total RNA was isolated as described above. RNA degradation and contamination were monitored using 1% agarose gels. The purity of the RNA was evaluated using Nanodrop 2000c (Thermo Fisher Scientific). Three biological replicates of RNA samples from each strain were pooled and subjected to RNA-Seq using the Illumina HiSeq 2000 platform (Novogene, China). Quality scores (Q20, Q30), GC content, and sequence duplication levels of the clean data were calculated (Additional file 2). All downstream analyses were performed using the high-quality clean data. Profiles of differentially expressed genes (DEG) of both strains were assessed using the DESeq R package (version 1.18.0), and analysis of variance was used to identify DEG between the strains. Adjusted *p* < 0.05 and log_2_ (fold change) values of 1 were set as thresholds for significantly differential expression levels. The sequencing data generated for this study were deposited in the SRA database under the accession number PRJNA947096.

### Western blot analysis

The presence of HIF-1α was assessed by Western blot analysis. Cell lysates were obtained by scraping the cells from IPEC-J2 cell line culture monolayers using SDS-PAGE buffer and sonication at 4 ℃ after 6 h incubation with CoCl_2_ in case of positive control and after 6 h of growth under normoxic and hypoxic conditions. Uniform sample concentrations (15 µL; based on cell number taken for the experiment) were run on an 8% gel using SDS-PAGE electrophoresis, at a constant voltage of 75 V for 20 min, then at 140 V for 1.5 h. The separated proteins were then transferred to a nitrocellulose membrane at a constant current (300 A) for 1.5 h. After 4 h blocking with 5% non-fat dry milk solution, the membranes were incubated with primary Ab [KO validated] HIF-1α Rabbit pAb (Abclonal, China) at a concentration of 1:1000 overnight in Tris-buffered saline (TBS) supplemented with 0.05% Triton X-100 (TBST) and washed with TBST (0.2%) four times for 10 min. The membranes were then treated with 1:2000 diluted secondary antibodies [Polyclonal Goat Anti-Rabbit (Dako, Denmark)] and incubated for 1 h. As a reference beta-Actin Ab (Cell Signaling) was used, with initial incubation for 1 h at a concentration of 1:2000. Western blotting was performed using Clarity Western ELC substrates (Bio-Rad).

### Statistical analysis

Data are presented as mean or geometric mean ± SEM in at least three independent experiments. Analysis was performed using GraphPad Prism software. Student *t*-test, one-way analysis of variance (ANOVA) with Tukey multiple comparison test, or Mann–Whitney U test was used to assess statistical significance. Differences were considered statistically significant at *p < 0.05, **p < 0.01 ***p < 0.001 or ****p < 0.0001.

## Results

### Low oxygen levels affect the adhesion of *Salmonella* Typhimurium

**Figure 1:**
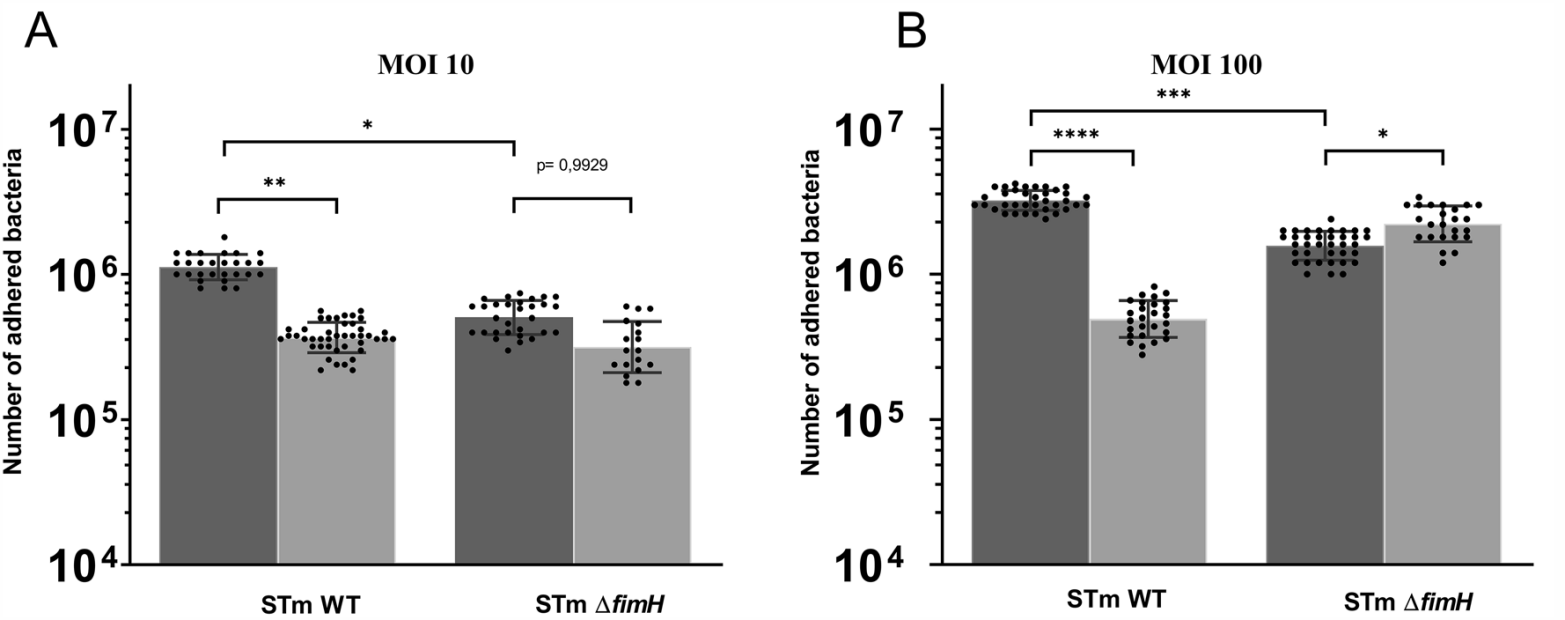
***Salmonella* Typhimurium and its *ΔfimH* mutant binding to IPEC-J2 cells in normal and low-oxygen conditions.** *S*TmWT and *S*Tm*ΔfimH* after the third passage were incubated for two hours with IPEC-J2 cell monolayers in normal oxygen level (dark gray bars) and low oxygen level (light gray bars). **A** MOI 10 **B** MOI 100; Statistical differences between strains were analyzed by one-way ANOVA with Tukey multiple comparison test, presented as geometric means with individual values indicated as dots. *p < 0.05, **p < 0.01, ***p < 0.001.

To investigate the impact of reduced oxygen levels on *Salmonella* Typhimurium adhesion, bacteria were grown under normal or low oxygen conditions. Growth curves under stationary growth conditions (without agitation) under normoxic and hypoxic conditions were obtained for WT and knockout strains. There were no differences in any of the typical growth phases, either between the WT and mutant or in the dependence on oxygen levels (Additional file 3A). Only *S*TmWT was able to agglutinate yeast cells, as *S*Tm*ΔfimH* does not produce mannose-dependent T1F (Additional file 3B). For adhesion assays, bacteria were passaged three times without shaking to induce high T1F expression [27]. As expected, adherence of *S*Tm*ΔfimH* was 2.5 times weaker at MOI 10 (*p* < 0.05) and 2.5 times weaker at MOI 100 (*p* < 0.001) compared to the WT strain (Figures 1A and B).

To investigate whether low oxygen levels present in the intestines affect T1F-dependent binding properties, we tested the adhesion of the IPEC-J2 cell line at normal and low oxygen concentrations. *Salmonella* Typhimurium and its mutant were grown under T1F-inducing conditions in normoxia or hypoxia, and their adhesion to the IPEC-J2 cells was measured in normoxic and hypoxic conditions. To confirm a low-oxygen phenotype of IPEC-J2 cells, we measured Hif-1α expression using mRNA and protein levels as markers of hypoxia. Although mRNA levels were similar in both oxygen levels, we noticed Hif-1α expression on the protein level only in hypoxia and control sample induced by CoCl_2_ (Additional file 4). Interestingly, the adhesion experiments with *Salmonella* Typhimurium T1F mutants at low oxygen concentrations revealed profound differences in their bacterial binding abilities (Figure 1).

At both tested MOI, the *S*TmWT strain exhibited significantly weaker binding under hypoxic conditions than under normoxic conditions. The amounts of adhered bacteria in hypoxia strongly decreased, approximately four times at MOI 10 (*p* < 0.01) and more than six times (*p* < 0.0001) at MOI 100, compared to that in normoxia. Notably, in the case of the mutant without T1F, the binding was not affected by low oxygen levels at MOI 10, and in the case of MOI 100 the mutant strain bound in higher amounts to IPEC-J2 cells compared to the WT strain (*p* < 0.05) in hypoxia. It is worth mentioning that these differences were not caused by different division rates of *Salmonella* throughout the experiment, because after incubating an equal number of bacteria corresponding to MOI 100 for 2 h under normal and low oxygen conditions, we did not observe any differences between the analyzed strains (Additional file 5).

### Differential gene expression in hypoxia and normoxia

**Figure 2:**
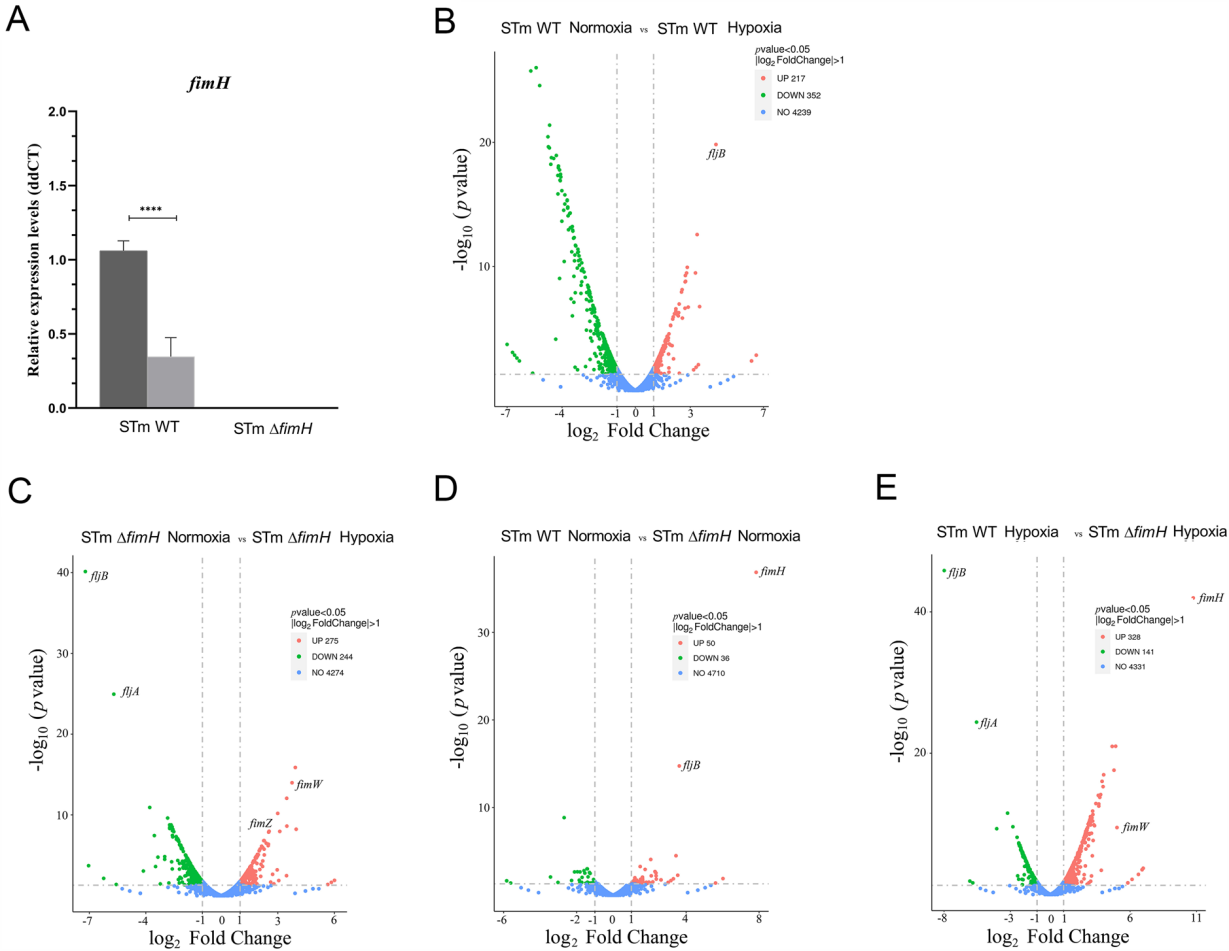
***Salmonella* Typhimurium gene expression under normoxia and hypoxia conditions. A**. *Salmonella’s fimH* gene expression. Relative expression of *fimH* mRNA in *S*TmWT and its mutant in normoxia (dark gray bars) and hypoxia (light gray bars) after the third passage of static growth in Luria–Bertani (LB) broth. Data represent the mean ± SD of three independent experiments. Triplicate samples were analyzed in each experiment to confirm the accuracy and reproducibility of qPCR. Statistical differences were analyzed by Student *t*-test (**A**) and are presented as the mean. ****p < 0.0001. **B**–**E** Vulcano plots for differentially expressed genes (DEG) in *S*TmWT *S*Tm*ΔfimH.* The green spots represent the downregulated genes, whereas the red spots represent upregulated genes. The blue spots indicate genes without significantly different expression levels between the two groups. *P* values for each point are indicated on the y-axis, lod2 of expression fold change indicated on the x-axis. **B** Comparison between *S*TmWT grown in normoxia versus hypoxia; **C** Comparison between *S*Tm*ΔfimH* grown in normoxia versus hypoxia. **D** Comparison between *S*TmWT *and S*Tm*ΔfimH* grown in normoxia; **E** Comparison between *S*TmWT *and S*Tm*ΔfimH* grown in hypoxia.

To verify the correlation between T1F expression and reduced adhesion under low-oxygen conditions, *fimH* mRNA expression levels were measured in bacteria grown under conditions that stimulated T1F expression. The findings indicate that relative expression of the *fimH* gene was significantly reduced under low-oxygen conditions in the *S*TmWT (*p* < 0.0001) (Figure 2A). As expected, in the case of *S*Tm*ΔfimH* no expression was detected.

Taking the above-mentioned results into consideration, we performed RNA sequencing (RNA-seq) using a high-throughput Illumina sequencing platform to investigate the gene expression profile of *S*TmWT and its *fimH* knockout grown under T1F-inducing conditions under normoxia and hypoxia. At least 14 500 000 clean reads (Additional file 2) were collected from each group. In total, 98.2% of clean reads were mapped to the reference genome in each group.

To define the bacterial transcriptome response to low oxygen levels in the presence or absence of T1F, we identified the related DEG. Global transcription profiles presented as volcano plots of DEG based on the threshold of fold change (|log_2_FC|> 1 and FDR < 0.05) are shown in Figures 2B–E. A total of 569 DEG were identified in *S*TmWT when compared under normoxic versus hypoxic conditions, among which 217 were upregulated and 352 were downregulated. When we compared *S*Tm*ΔfimH* strain using the same conditions, 519 DEG were identified, among which 275 were upregulated and 244 were downregulated.

Significant DEG were sorted employing fold changes, and genes known to be involved in adhesion, invasion, and motility were selected (Additional file 6). When we compared *S*TmWT DEG between normal and low oxygen conditions, we observed upregulation of effectors of the T3SS apparatus, plasmid-encoded fimbria genes, and notably, flagellins and flagella regulatory genes in normoxia. By contrast, genes encoding some fimbrial structures (elements of *lpf*, *stf*, *bcf*, *sti* operons, and Type IV fimbriae) were downregulated in normoxia. When we compared *S*Tm*ΔfimH* DEG in normoxia and hypoxia under T1F-inducing conditions, a majority of downregulated genes in normoxia were related to the flagellar system, whereas upregulated genes encoded elements of the T3SS, T1F operon, and elements of *stb* and *std* fimbrial operons.

Among all the analyzed DEG, expression of the *fljB* gene encoding phase II flagellin was significantly reduced in the low oxygen environment in *S*TmWT. Interestingly, in *S*Tm*ΔfimH*, *fljB* gene expression level was higher in hypoxia than in normoxia, which agreed with the adhesiveness of this strain. Bearing that in mind, we investigated the motility of both the strains under normal and low oxygen conditions and validated the expression levels of genes related to flagellins, motility, and T1F-dependent adhesion.

### Hypoxia negatively impacts flagella- and motility-related gene expression level

To verify the effect of hypoxia on *Salmonella’s* adhesion and motility, selected genes were subjected to qPCR analysis.

**Figure 3:**
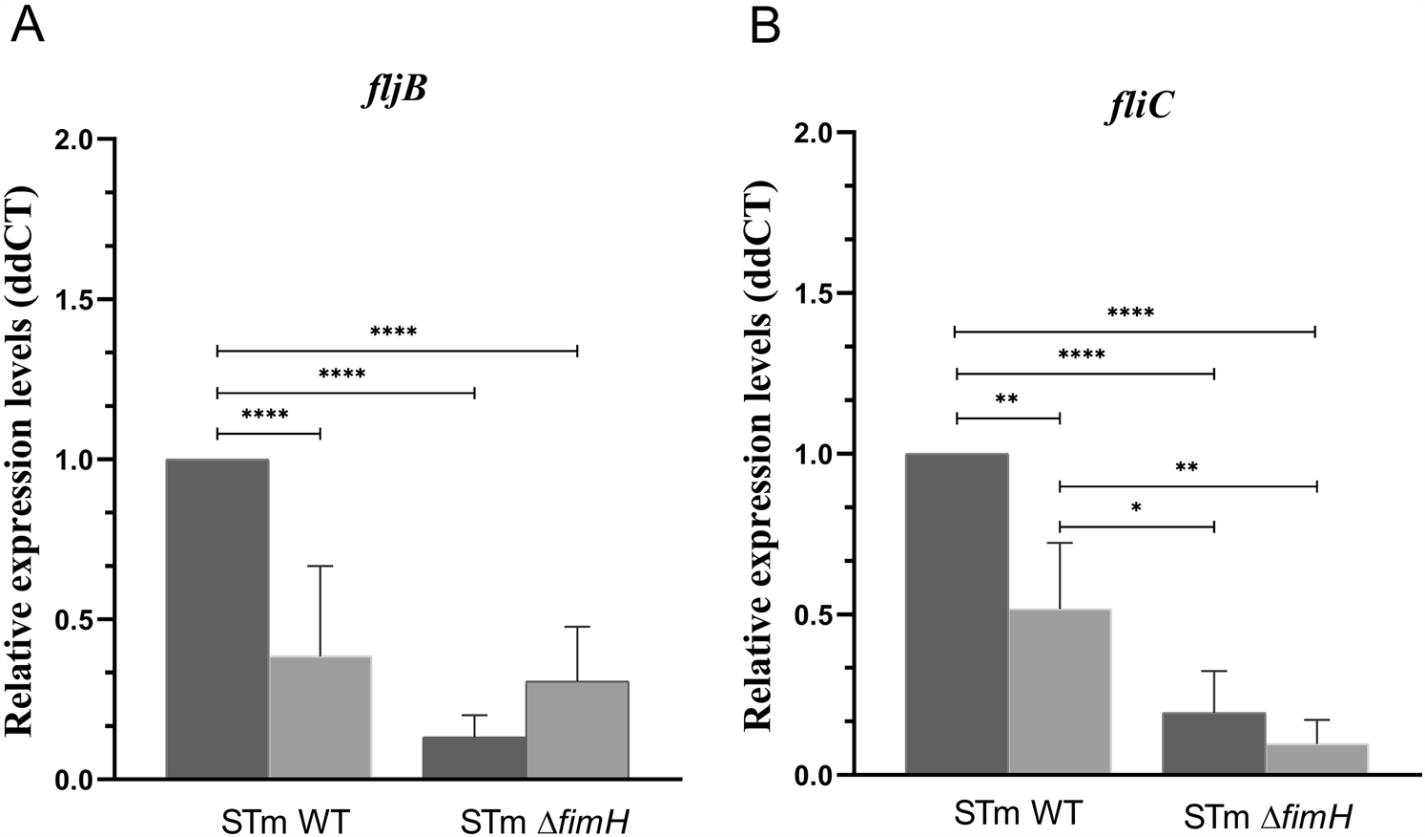
***Salmonella’s* flagellin*s genes* expression.** Relative expression of *fljB* (**A**) and *fliC* (**B**) mRNA in *S*TmWT and *S*Tm*ΔfimH* in normoxia (dark gray) and hypoxia (light gray) after third passage during static growth in Luria–Bertani (LB) broth. Data represent mean ± SD of three independent experiments. Triplicate samples were analyzed in each experiment to confirm the accuracy and reproducibility of qPCR. Statistical differences between strains were analyzed by one-way ANOVA with Tukey multiple comparison test, presented as geometric means with individual values indicated as dots. *p < 0.05, **p < 0.01, ****p < 0.0001.

When we analyzed the expression levels of two genes encoding *Salmonella* flagellins (*fljB* and *fliC*), we observed that in the *S*TmWT strain expression of both genes was significantly decreased under hypoxic conditions (*p* < 0.0001 and *p* < 0.01, respectively) (Figure 3). Interestingly, the oxygen level did not significantly affect *fliC* expression and caused higher expression of *fljB*, but in both cases, the mutant strain expressed flagellins at significantly lower levels than the WT strain. Different flagellar expression patterns depend not only on oxygen levels but also on the presence or absence of T1F, suggesting that the cross-talk between these structures may depend on local oxygen concentration. Therefore, we evaluated the expression of genes previously found to be involved in T1F and flagella cross-talk (*fimZ*, *flhD*, *fliA*, *fliZ*) [2, 28].

**Figure 4:**
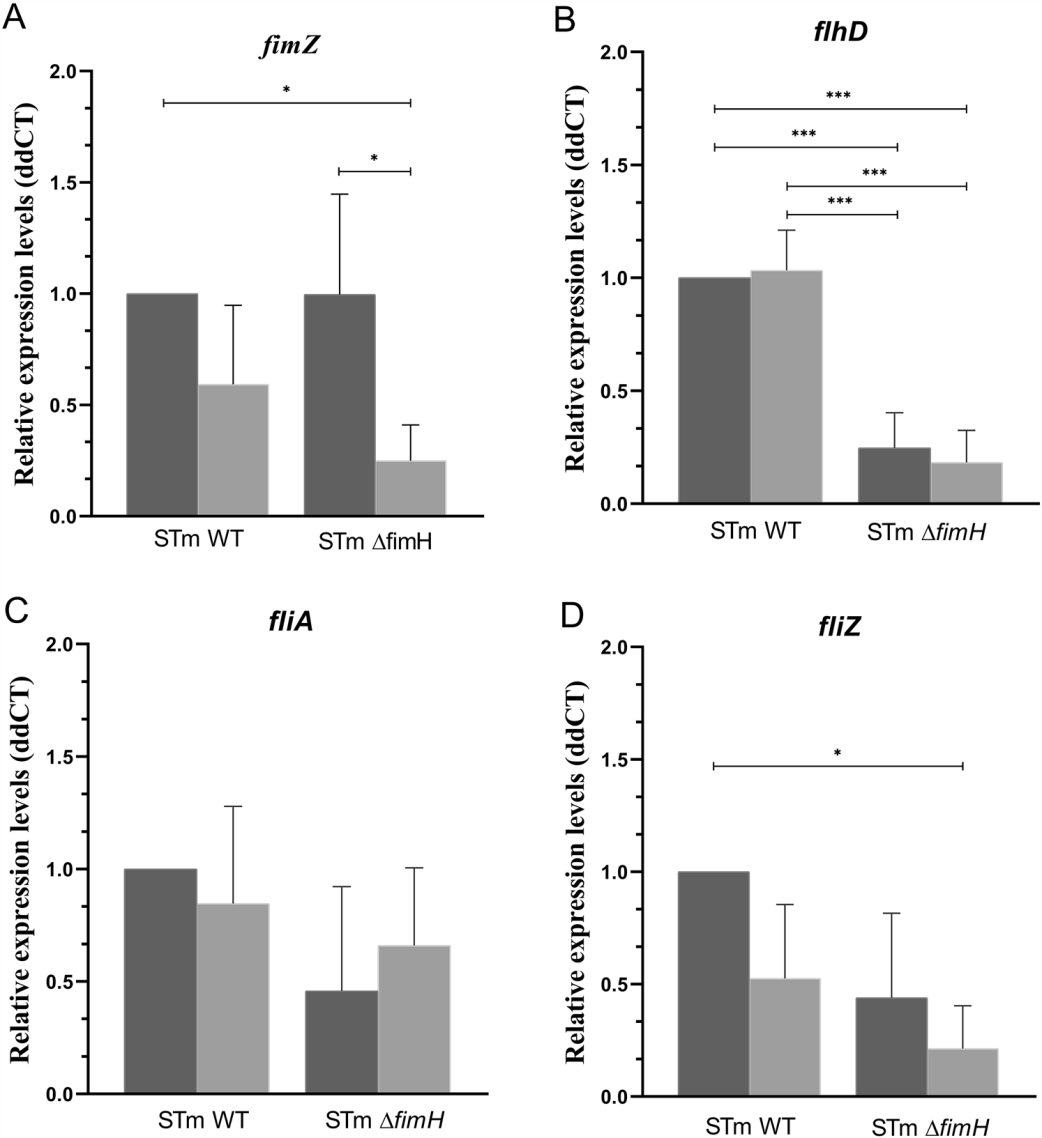
**Relative expression of *Salmonella* Typhimurium genes involved in flagella-T1F cross-talk in hypoxia.** Relative expression of **A** *fimZ*, **B** *flhD*** C** *filA*, and **D** *fliZ* mRNA in *S*TmWT and *S*Tm*ΔfimH* in normoxia (dark gray bars) and hypoxia (light gray bars) after the third passage during static growth in Luria–Bertani (LB) broth. Data represent the mean ± SD of three independent experiments. Statistical differences between strains were analyzed by one-way ANOVA with Tukey multiple comparison test, presented as geometric means with individual values indicated as dots. *p < 0.05, ***p < 0.001.

Lowered oxygen levels caused a significant decrease in *fimZ* gene expression in the *ΔfimH* mutant (*p* < 0.05) and a tendency to decrease, but not a significant change, in *S*TmWT (*p* = 0.06) (Figure 4A). In the next step, we aimed at investigating the differences in the expression of the *flhD* gene, a regulator of flagellar biosynthesis. We observed no significant differences in its expression between normoxic and hypoxic conditions (Figure 4B). However, there was a significant decrease in the expression level of *flhD* between *S*TmWT compared to *S*Tm*ΔfimH* at both oxygen levels (*p* < 0.001).

Analysis of expression of the *fliA* gene revealed that changes in oxygen availability did not influence its expression in either of the strains analyzed (Figure 4C). Moreover, there was no significant difference in the expression between the WT and mutant strains. In the case of the *fliZ* gene, the *S*TmWT strain exhibited significantly lower expression under hypoxic conditions (*p* < 0.05). Interestingly, the expression of *fliZ* was significantly lower in *S*Tm*ΔfimH* than in the WT strain, with no differences seen related to the oxygen levels (Figure 4D).

### Hypoxia decreases motility of *Salmonella* Typhimurium

**Figure 5:**
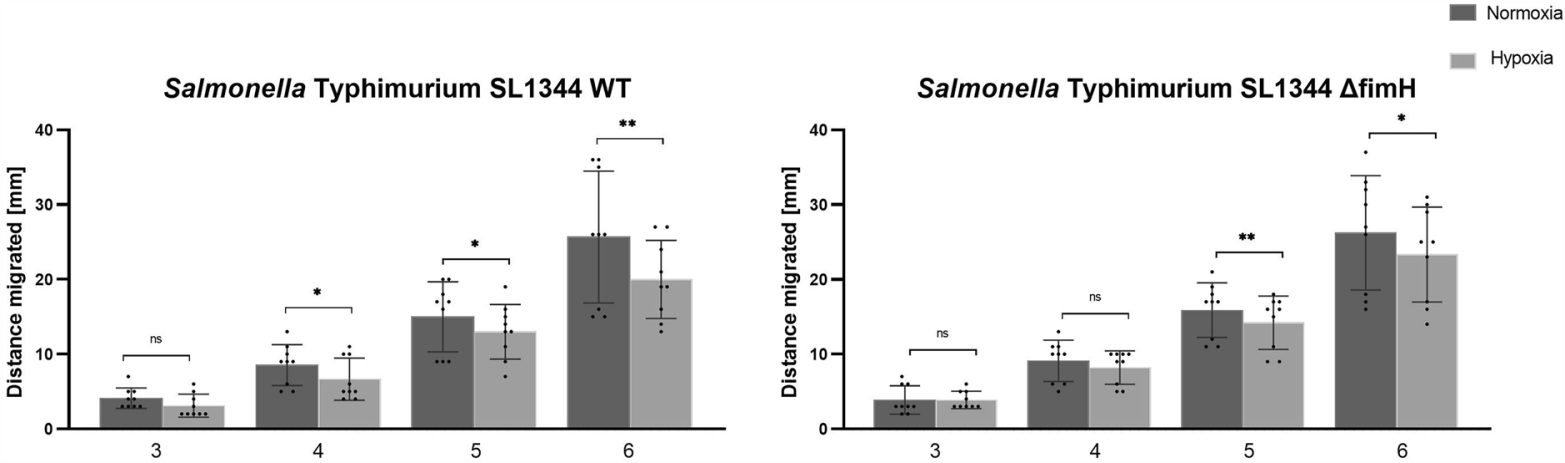
**Migration assays of *Salmonella* Typhimurium.** *S*TmWT (**A**) and *S*Tm*ΔfimH* (**B**) after the third passage were assessed for motility in soft agar at normal oxygen level (dark gray bars) and low oxygen level (light gray bars). Statistical differences between strains were analyzed by the Student *t*-test, and presented as mean values; *p < 0.05; **p < 0.01.

Considering that flagellin gene expression was significantly decreased due to low oxygen levels under T1F-inducing conditions, we investigated whether and how hypoxia affects the *Salmonella* motility phenotype. First, we investigated the migratory behavior of the *Salmonella* strains. Bacteria were grown under T1F-inducing conditions and then spotted on LB plates with 0.3% agar. The distance of migration was measured at 3, 4, 5, and 6 h (Figure 5).

In the case of *S*TmWT, the migration radius was significantly smaller under hypoxic conditions, with noticeable differences in the mean migratory radius after 4, 5, and 6 h. In the case of *S*Tm*ΔfimH* the migration radius was smaller and significant only after the 5^th^ and 6^th^ hour.

**Figure 6:**
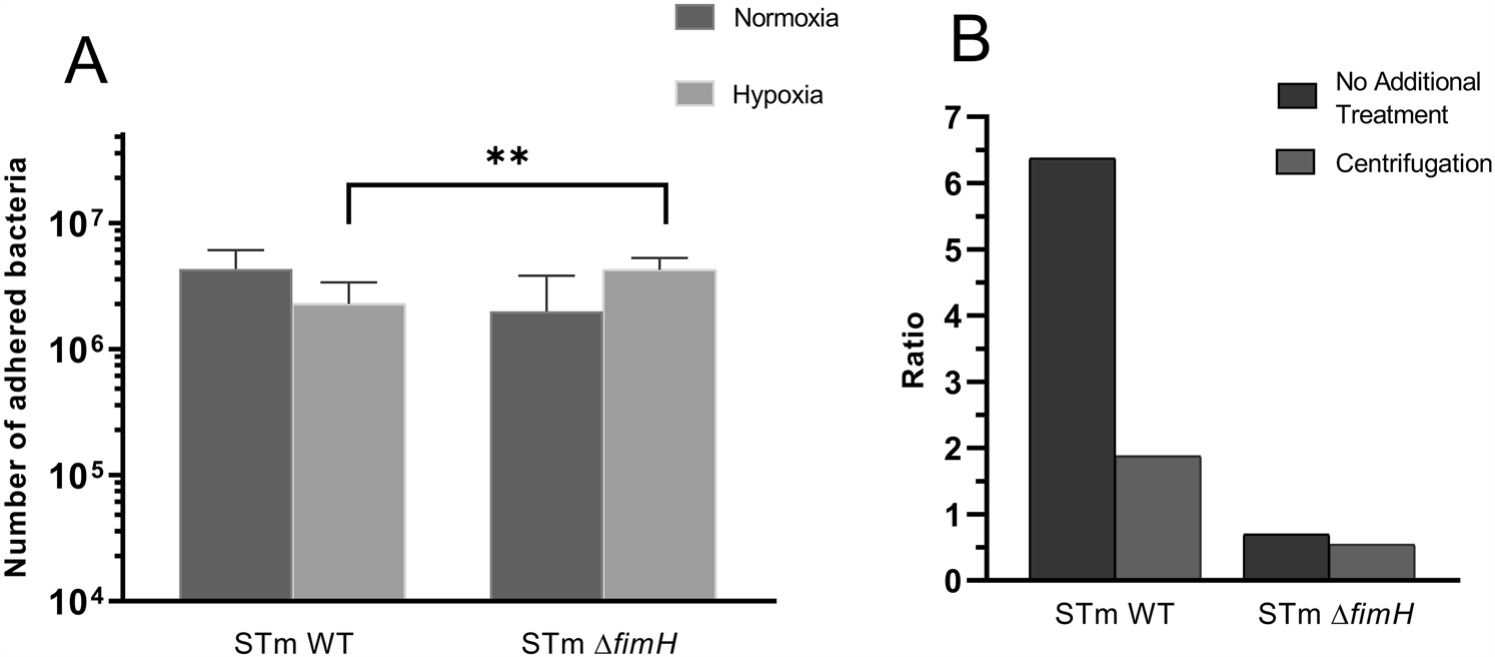
**Binding of* Salmonella* Typhimurium to IPEC-J2 cells under normal and low oxygen conditions after centrifugation. A** *S*TmWT and *S*Tm*ΔfimH* after the third passage were forced to faster contact with IPEC-J2 cells using centrifugation. Cell monolayers at normal oxygen level (dark gray bars) and low oxygen level (light gray bars) at MOI 100. **B** Ratio of adhesion level (normoxia/hypoxia) with (light bars) and without (dark bars) centrifugation. Statistical differences between strains were analyzed by one-way ANOVA with Tukey multiple comparison test presented as individual values with a geometric mean; there were no statistically significant differences.

To investigate the impact of motility on *Salmonella* adhesion profiles, we established faster and direct contact between bacteria and cells using centrifugation (Figure 6).

As expected, the adhesion level was significantly higher after centrifugation when compared to non-centrifugated bacteria (Figure 6A). Importantly, the difference between the adhesion levels in normoxia and hypoxia significantly decreased after centrifugation. (Figure 6B). For *S*TmWT, the ratio of the adhesion level in normoxia to that in hypoxia decreased from 6.37 (without centrifugation) to 1.88 (after centrifugation). Nevertheless, in the case of the *S*Tm*ΔfimH* mutant, we did not observe distinct changes (0.70 without centrifugation to 0.54 after centrifugation). It is worth mentioning that even with forced contact, there was a significant difference in binding between *S*TmWT and *S*Tm*ΔfimH* (two fold, *p* < 0.01).

### Salmonella biofilm formation affected by hypoxia

Studies have confirmed that flagellum-driven motility is crucial during the early stages of biofilm formation on biotic and abiotic surfaces [13]. Hypoxia impairs the motility of *Salmonella* and influences the expression of flagellum-related genes. Therefore, we determined the influence of oxygen levels on the biofilm-forming ability of *S*TmWT and *S*Tm*ΔfimH.*

**Figure 7:**
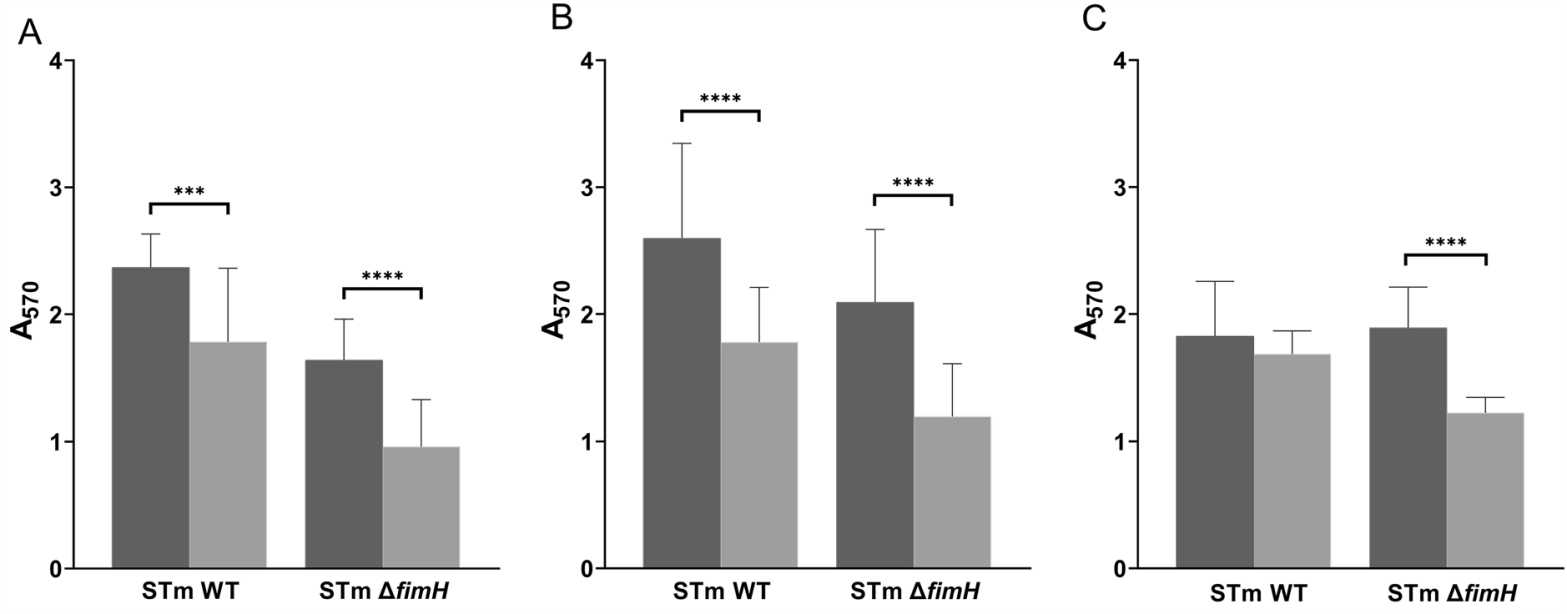
**Biofilm formation abilities of *S*TmWT and *S*Tm*ΔfimH* for A: 24, B: 48, and C:72 h in normoxic (dark gray bars) and hypoxic (light gray bars) conditions.** The columns represent mean ± standard deviation. Statistical significance analysis was performed using the Mann–Whitney test.***p < 0.001;****p < 0.0001.

**Figure 8:**
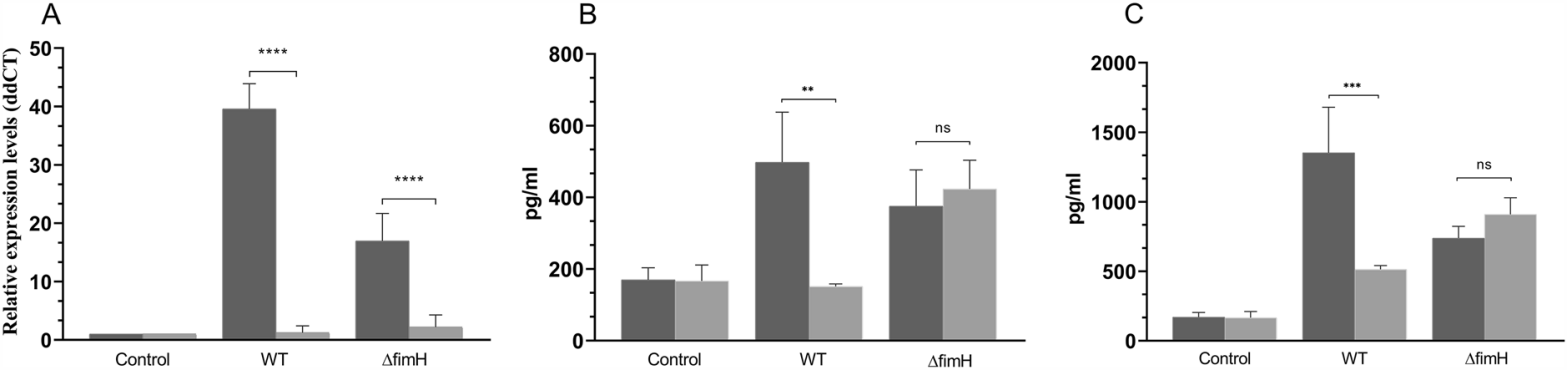
**IL-8 expression and secretion during *Salmonella* Typhimurium infection. A** Expression of *Il-8* mRNA in IPEC-J2 cells following 2 h of infection with MOI 100 of *S*TmWT and *S*Tm*ΔfimH* after the third passage in normal oxygen level (dark gray bars) and low oxygen level (light gray bars). Non-infected IPEC-J2 cells were assigned as a calibrator sample, and fold change was measured with unstimulated cells set at 1. Data represent mean ± SD of at least three independent experiments. Triplicate samples were analyzed in each experiment to confirm the accuracy and reproducibility of qPCR. **B**, **C** Secretion of IL-8 by IPEC-J2 cells exposed to *S*TmWT and *S*Tm*ΔfimH* after the third passage in normal oxygen level (dark gray bars) and low oxygen level (light gray bars). After 2 (**B**) or 4 (**C**) h of incubation, secretion of IL-8 was measured by ELISA. Interleukin levels were normalized against unstimulated cells. Data represent mean ± SD of four independent experiments and were analyzed in each experiment to confirm the accuracy and reproducibility of ELISA. Statistical differences between strains were analyzed by one-way ANOVA with Tukey multiple comparison test, presented as geometric means with individual values indicated as dots. ^ns^ non-significant; *p < 0.05, **p < 0.01; ***p < 0.001.

Biofilm formation was assessed after 24, 48, and 72 h of culturing under normoxic and hypoxic conditions. The differences in biofilm formation abilities are shown in Figure 8, where the crystal violet absorbance corresponds to the formed biofilm biomass. *S*TmWT and *S*Tm*ΔfimH* demonstrated a significant reduction in biofilm production capacity under lower oxygen growth conditions after 24 and 48 h of culture (Figures 7A and B). Such differences were not observed for the WT strain at 72 h of culture, because we could not observe any significant changes in biofilm formation at this time point. Only the *S*Tm*ΔfimH* mutant was unable to reach the biofilm production level in hypoxia such as in normoxia conditions, even after 72 h of culture. These results indicate that hypoxia impairs biofilm formation, particularly during the initial stages of this process.

### Hypoxia strongly decreases IL-8 expression and secretion induced by *Salmonella* infection

It has previously been shown that flagellin expression may induce pro-inflammatory cytokine expression via the activation of TLR5 [29, 30]. Therefore, we decided to measure the expression and secretion level of IL-8 as representative proinflammatory mediators. Cells were infected with *S*TmWT and *S*Tm*ΔfimH* for 2 h (mRNA) or 2 and 4 h (protein), and the level of *Il-8* mRNA and IL-8 secretion were measured. As expected, *Il-8* mRNA expression was strongly increased during the *Salmonella* infection and was significantly higher in the WT strain than in the mutant strain (Figure 8A). The results obtained for the mRNA level were confirmed at the protein level only in the case of *S*TmWT, in which IL-8 was produced at a significantly higher level in the supernatant after 2 and 4 h of infection at normal oxygen concentrations compared to that in uninfected cells (Figures 8B and C). Interestingly, under hypoxic conditions, the IL-8 secretion level after 2 h was comparable to that in uninfected cells, and after 4 h post-infection, we also noted an increase in IL-8 secretion in hypoxia. However, this change was significantly smaller than that observed in the WT strain. When cells were infected with the *S*Tm*ΔfimH* strain, there was no difference in IL-8 secretion between hypoxic and normoxic conditions; however, in both cases, secretion of cytokine levels was significantly higher than in uninfected cells.

## Discussion

During infection, invading enteropathogens are exposed to harsh and challenging environmental conditions in the host intestines. In addition to the host immunological defense mechanisms, the pathogens compete for environmental niches, energy, and carbon sources with billions of microbes inhabiting the host intestines. The gut microbiome appears to be a critical factor that plays a role in resistance of enteric pathogen colonization; however, the exact mechanism has not been fully elucidated [31]. The intestinal microbiome occupies the environmental niches and limits oxygen availability [32]. Oxygen level dynamics play a pivotal role in intestinal homeostasis and low availability of oxygen, as well as its gradient, supports diverse intestinal microbiota and limits the spread of potential intestinal pathogens. It has been observed that the partial pressure of oxygen rapidly decreases along the radial axis from the intestinal submucosa to the lumen [33, 34]. Therefore, modification of culture and experimental conditions by changing oxygen availability may influence biochemical and biophysical processes during pathogen infection in vitro and more accurately mimic the intestinal environment. Interestingly, when host–pathogen interactions are investigated, oxygen availability in the gastrointestinal tract is often overlooked [35]. Previous studies have identified oxygen as a critical resource for *Salmonella* infection and intestinal colonization [20, 36]. *Salmonella* can not only grow in both hypoxic and normoxic conditions, but can also manipulate local oxygen levels to outcompete the microbiota. Although hypoxia affects host–pathogen interactions in multiple ways, the role of local oxygen levels in *Salmonella* infections is still unclear.

In this study, we took a closer look at the initial stages of *Salmonella* infection, with an emphasis on adhesion of *Salmonella* to host cells in conditions of limited oxygen availability. The first step in its pathogenesis is adherence to intestinal epithelial cells (IEC) via specific receptors, followed by colonization of the intestinal surface. Attachment to the intestinal mucosa is driven by adhesive structures like fimbrial and non-fimbrial adhesins, flagella or elements of Type 3 Secretion Systems. Among the fimbrial operons in the *Salmonella* genome [37], the type 1 fimbriae (T1F) encoded by the *fim* operon are among the most extensively studied [3]. A lectin-like protein called FimH is directly responsible for the binding properties [8, 38]. The role of T1F in adhesion of *Salmonella* to eukaryotic cells as well as in *Salmonella* pathogenesis has been investigated using various cell and animal models (reviewed in [3]). Therefore, we investigated the effect of low oxygen concentrations on the T1F-dependent adhesion of *Salmonella* Typhimurium. In our study, *Salmonella* Typhimurium SL1344 shows significantly lower adhesion in conditions of reduced oxygen availability. These differences, at least partially, may be related to the significantly lower expression of the *fimH* gene and, therefore, T1F in hypoxia. A mutant with no T1F did not reduce the binding level in hypoxia, and a comparison between the lowest adhesion levels suggested that under the tested conditions, there was a minimum binding level of approximately 2 × 10^5^ CFU, e.g., one bacterium per cell, which was completely independent of MOI, presence of T1F, or oxygen levels. This minimal binding is driven by various adhesion systems and virulence factors. Along with adhesive structures, such as T1F, other systems, especially SPI-1, T3SS, and flagella, have been implicated in *Salmonella* pathogenesis. Multiple studies have shown intensive crosstalk between these systems [2, 39, 40]. However, their dynamics, especially under hypoxic conditions, remain relatively unknown. Our results show that reduced *Salmonella* adhesion levels under low-oxygen conditions may be related to reduced motility of *Salmonella* caused by altered flagellin expression. *Salmonella* Typhimurium can express two flagellin monomers such as FliC (flagellin phase type 1/H1) and FljB (flagellin phase type 2/H2); their expression is regulated by a phase variation mechanism, with only one flagellin expressed at a time [41, 42]. There were no clear differences in the normal swimming motility of FliC- or FljB-expressing bacteria; however, it has been observed that, under high-viscosity conditions, FljB + *Salmonella* swim more efficiently [43].

In the current study, we noticed that in bacteria grown under T1F-inducing conditions [27], expression of both the flagellins significantly decreased at low oxygen levels. Importantly, the *S*Tm*ΔfimH* strain expressed *fljB* and *fliC* at a significantly lower level than the WT strain in hypoxia. These results suggest that T1F expression affects not only the adhesion, but also the motility of *Salmonella* Typhimurium, especially at low oxygen levels. Our data show that hypoxia reduced the motility *of Salmonella* which was confirmed by a motility assay in soft agar. Moreover, the radius of *S*Tm*ΔfimH* migration was smaller and significant only after the 5th and 6th hour of the assay, which corresponded directly with *fljB* and *fliC* expression levels.

Flagella-expressing bacteria can move through an environment by means of the movement of flagella, which is important for various processes such as finding nutrients, avoiding toxins, and colonizing host tissues. Bacterial movement is regulated by a complex network of genetic and biochemical mechanisms that control the assembly, function, and regulation of molecular motors and structures responsible for motility. It was previously shown that the major regulatory elements for T1F is the *fimZ* gene and for the flagellar system *flhD*, *fliA*, and *fliZ* [2, 3, 28]. FimZ, a major T1F activator, increases T1F expression by activating the *fimA* promoter, at the same time negatively affecting flagellar expression [2, 3, 28]. In our study, we show that in hypoxia, *fimZ* expression was decreased (*p* < 0.06) in the WT strain, and significantly lower (*p* < 0.05) in the mutant strain, which corresponds to lower *fimH* expression in the WT strain. It has been previously shown that deletion or lower expression of *fimZ* makes *Salmonella* Typhimurium supermotile [44]. Surprisingly, despite decreased *fimZ* expression in hypoxia, we observed significantly reduced motility of *S*TmWT as well as its *ΔfimH* mutant. Among the flagellar regulators, *fliZ* was previously implicated in T1F flagellar crosstalk [2]. Here, we observed that *fliZ* expression was decreased in hypoxia in T1F-inducing conditions in both the WT and mutant strains.

Taking into account the fact that in conditions of limited oxygen availability, both *fimZ* and *fliZ* gene expression was reduced together with a reduction of expression of *fljB* and *fliC* genes, thus affecting not only T1F-dependent adhesion, but also the motility behavior of *Salmonella*, we speculate that hypoxia affects T1F-flagella cross-talk in a different way than was previously shown in normoxia. Both the flagella and T1F are regulated at multiple levels in a very complex manner, starting from transcription, translation, structural assembly, and ending with stability. Under our experimental conditions, the two systems responsible for adhesion and motility may have been negatively affected by low oxygen availability. Notably, the lack of *fimH* expression in the mutant strain did not affect the *fimZ* expression level, but significantly decreased the expression of the major flagella regulators, the *flhD* and *fliZ* genes.

To confirm that reduced adhesion in hypoxia is caused directly by reduced motility, in agreement with results related to RNA-Seq, qPCR, and motility in soft agar, we established a direct contact of the bacteria with host cells by centrifugation prior to performing the adhesion test. Previous studies have suggested that centrifugation can restore the reduced ability of non-flagellated *Salmonella* Typhimurium to infect cells during infection and that differences in adhesion due to motility defects can be compensated for by centrifugation [6]. We found that the differences in binding under normoxic and hypoxic conditions observed in standard adhesion assays disappear after centrifugation. This result supports the idea that the major cause of altered adhesion behavior in hypoxia is reduced motility of *Salmonella.* Importantly, despite higher binding levels in all the analyzed strains and conditions, we were still able to detect significantly important differences between *S*TmWT and *S*Tm*ΔfimH* strains*.* This shows that, even with the limited role of motility in adhesion tests, T1F strongly impacts the host-microbe–binding phenotype.

In addition to motility, flagella play a role in biofilm formation and stimulation of the innate immune response [5, 45]. When we investigated the biofilm-forming ability of *Salmonella* Typhimurium in our T1F-induced model under normal and limited oxygen conditions, significant differences were detected. In the WT strain, there was higher biofilm production in normoxia rather than in hypoxia at all examined time points. In *S*Tm*ΔfimH*, after 24 and 48 h, biofilm production in hypoxia was lower than in normoxia, which again correlates with flagella gene expression. Importantly, there were significant differences in biofilm formation levels between *S*TmWT and *S*Tm*ΔfimH,* which provided evidence that not only flagella but also T1F plays a significant role in biofilm formation. Several studies have shown that various types of fimbriae are crucial in enabling specific bacteria to form biofilms [46–48]. However, their effect on bacterial attachment and biofilm growth depends on the type of bacteria and fimbriae. For example, type 3 fimbriae strongly promote biofilm development in *Klebsiella pneumoniae* [46]. In contrast, *E. coli* biofilm development is impeded when type 1 fimbriae are suppressed because they are essential for irreversible cell attachment and subsequent biofilm formation, but not for the initial reversible cell attachment. Therefore, our results highlight that biofilm systems have highly complex dynamics and show that T1F expression seems to promote biofilm formation in *Salmonella* Typhimurium in correspondence with flagella and oxygen levels.

Flagellins induce an immune response via TLR activation, which results in proinflammatory cytokine expression [29, 49]. Flagellin-induced TLR5-mediated gene expression protects the host from pathogens such as *Salmonella* [50]. In the current study, we selected IL-8 as a representative proinflammatory mediator because of the known relationship between flagellin and pro-inflammatory cytokine expression [30, 51]. It was previously shown that Caco-2 cells stimulated by *Salmonella* Typhimurium flagellin produce IL-8 in a dose-dependent manner [29]. During the initial stages of *Salmonella* infection, IL-8 plays a fundamental role in the initiation and mediation of innate immune responses by recruiting neutrophils, lymphocytes, and eosinophils to the infection sites [52]. In our model, IL-8 expression and secretion were strongly upregulated during *Salmonella* infection. However, at reduced oxygen levels, there were no differences between the non-infected cells and those infected with *Salmonella*. This neutralization of IL-8 expression level may have been caused either by the lowered flagellin expression as mentioned above or by the impact of HIF1-α stabilized in hypoxia [53]. In our experiments, we noticed increased HIF1-α stability in hypoxia on protein level, despite no changes in mRNA level. It is worth mentioning, that the majority of commercially available antibodies do not correctly recognize porcine HIF1-α.

HIF1-α may also act as a regulator of the intestinal barrier integrity [18, 35] by providing a barrier-protective mechanism in the intestines [19, 25]. The upregulation of HIF1-α under hypoxic conditions may impact *Salmonella's* binding and infection by affecting tight junctions, which serve as entry sites for the bacteria. Conversely, previous research has demonstrated that *Enterobacteriaceae* infection can also activate HIF-1 and alter epithelial cells, despite the fact that HIF1-α does not affect proinflammatory gene expression in IEC [54].

We demonstrate that low oxygen levels usually found in *Salmonella*-infected gut tissue may significantly affect the initial stages of *Salmonella* infection. First, the analysis of *Salmonella*-host interactions under low oxygen conditions may uncover key features of pathogenesis that have been overlooked in previous studies conducted under normoxic conditions. For example, *Salmonella*’s response to low-oxygen conditions may enable it to evade host immune responses and promote bacterial survival and replication in the host. Second, targeting adaptation and responses to hypoxic conditions of pathogens may be a promising approach for development of new treatment strategies for *Salmonella* infection. One potential strategy is to increase local oxygen levels in infected tissues to inhibit bacterial growth and enhance host immune responses. Another approach is to develop drugs that specifically target *Salmonella*’s hypoxic response pathways, such as those involving HIF-1α or other transcription factors that are activated in response to low oxygen levels. In addition, understanding the role of low oxygen tension in the gut may have broader implications for the treatment of other gastrointestinal infections caused by bacteria that thrive under hypoxic conditions, such as *Clostridium difficile* and *Helicobacter pylori*. Overall, these findings highlight the importance of the impact of hypoxia on *Salmonella* pathogenesis and suggest new avenues for the development of targeted and effective treatments for *Salmonella* and other bacterial infections.

### Supplementary Information

**Additional file 1. Primers used in this study.** List of all primers for PCR and qPCR used in this study;

**Additional file 2. RNA-Seq quality**. Analysis of the data quality for RNA-Sequencing; The Q20, Q30, GC-content, and sequence duplication level data.

**Additional file 3. *Salmonella* strains characterization – growth curves. A**. Growth curves in stationary growth conditions (without agitation) in normoxia and hypoxia (1% of oxygen) were obtained for all the generated mutants. **B**. Agglutination assay. Detection of T1F expression by microplate yeast agglutination test. The numbers indicate the geometric average of the titer endpoint (*N* = 5) for both *Salmonella* strains.

**Additional file 4. Hif-1α expression. A**. Expression of Hif-1α mRNA in IPEC-J2 cell line following 2 h infection with MOI 100 of *Salmonella* Typhimurium wild type (*S*TmWT) and T1F *S*TmΔfimH) after the third passage in normal oxygen level (dark gray bars) and low oxygen level (light gray bars). Non-infected IPEC-J2 cells were assigned as a calibrator sample, and fold change was measured over unstimulated cells set at 1. Data represent the mean ± SD of at least three independent experiments. Triplicate samples were analyzed in each experiment to confirm the accuracy and reproducibility of qPCR. Statistical differences between strains were analyzed by Student *t*-test, and presented as individual values with a mean; there were non-significant differences between the samples. **B**. Western blot of IPEC-J2 cells grown in hypoxia, normoxia or in the presence of CoCL2. The presence of HIF-1α was assessed by Western blot analysis in IPEC-J2 cell lysates obtained by scraping the cells using SDS-PAGE buffer and sonication at 4 ℃ after 6 h incubation with CoCl2 and after 6 h of growth under normoxic and hypoxic conditions. As a reference beta-Actin Ab (Cell Signaling) was used.

**Additional file 5. *Salmonella* Typhimurium 2 h incubation without cells.** *Salmonella*. Typhimurium wild type (STmWT) and T1F mutants STmΔfimH (after the third passage were incubated for two hours in an infection medium in 24-well plates in normal oxygen level (dark gray bars) and low oxygen level (light gray bars).

**Additional file 6. DEG (Differentially expressed genes).** Comparison of differentially expressed genes involved in adhesion, invasion, and motility of *Salmonella* Typhimurium WT and Δ*fimH* mutant.

**Additional file 7. Stability of the expression of 16S reference.** Stability of the expression of 16S reference gene in analyzed STm strains (WT and Δ*fimH* mutant) in both growth conditions (normoxia and hypoxia). The average Ct values within all biological replicates were around 10 and fluctuated less than one cycle (10.44 ± 0.74, 10.40 ± 0.57, 10.58 ± 0.6, 10.21 ± 0.73, respectively).

## Data Availability

The datasets generated and/or analyzed during the current study are available in the SRA database, under the accession number PRJNA947096. All the other datasets used and/or analyzed during the current study are available from the corresponding author on reasonable request.
