# Supplementary material for: Salmonella adhesion is decreased by hypoxia due to adhesion and motility structure crosstalk
Source: Vet Res. 2023 Oct 24;54:99. doi: 10.1186/s13567-023-01233-2 (PMC10598919; doi:10.1186/s13567-023-01233-2)
Supplement: Supplementary file 1 — Additional file 1. Primers used in this study. List of all primers for PCR and qPCR used in this study; [file 13567_2023_1233_MOESM1_ESM.docx]

**Additional file 1 Primers used in this study.**

|  |  | Tm | Length |
| --- | --- | --- | --- |
| fimH_F | TCATGGGCGTCGACTATAAC | 51,8 |  |
| fimH_R | CGAGGGATCGTCACCATATT |  |  |
| fimW_F | AACTGATCGCCAGCGGTTAT | 60 | 80 |
| fimW_R | GCGGAGTGACTTTGTCTGCT |  |  |
| fimZ_F | GTTACCGTGCTGCGCTATCT | 60 | 95 |
| fimZ_R | GCCTTATGCGCGCTGATAGT |  |  |
| flhD_F | AGATGGTCAAACTGGCGGAG | 60 | 92 |
| flhD_R | GCGCGAATCCTGAGTCAAAC |  |  |
| fliA_F | TCAAAGAGATTGGCGCGGTA | 60 | 91 |
| fliA_R | CCCAGTTTGGTGCGTAATCG |  |  |
| fliC_F | CTGCTTTGGCACAGGTTGAC | 60 | 84 |
| fliC_R | TGCCCAGGTTGGTAATAGCG |  |  |
| fliZ_F | GCCGGAAACCAGCACCAATA | 60 | 105 |
| fliZ_R | CTGGCGGTAAAGGGGGATTT |  |  |
| fljB_F | GTGGTACGAATGGTACGGCT | 60 | 98 |
| fljB_R | AGCACCAGTAAAGCCACCAA |  |  |
| Hif-1α_F | TGCCAGAACCTCCTGTAACC | 58 | 125 |
| Hif-1α_R | ATGTACGTGGGGAGGAGATG | 58 |  |
| F_seq | GGCGATTACGATAGCCAGCGC | 60 |  |
| R_seq | CAGCGGGCTGAACAAAACACAAC | 60 |  |
