# Supplementary material for: Salmonella adhesion is decreased by hypoxia due to adhesion and motility structure crosstalk
Source: Vet Res. 2023 Oct 24;54:99. doi: 10.1186/s13567-023-01233-2 (PMC10598919; doi:10.1186/s13567-023-01233-2)
Supplement: Supplementary file 2 — Additional file 2. RNA-Seq quality. Analysis of the data quality for RNA-Sequencing; The Q20, Q30, GC-content, and sequence duplication level data. [file 13567_2023_1233_MOESM2_ESM.docx]

| **Sample name** | **Raw reads** | **Clean reads** | **Raw bases** | **Clean bases** | **Error rate (%)** | **Q20 (%)** | **Q30 (%)** | **GC content (%)** |
| --- | --- | --- | --- | --- | --- | --- | --- | --- |
| SL1344_WT_Nb | 18120702 | 17740710 | 2.7G | 2.7G | 0.02 | 98.28 | 94.94 | 53.39 |
| SL1344_D_Nb | 17533122 | 17201380 | 2.6G | 2.6G | 0.02 | 98.23 | 94.82 | 53.37 |
| SL1344_WT_Hb | 17266936 | 16892024 | 2.6G | 2.5G | 0.02 | 98.38 | 95.17 | 53.32 |
| SL1344_D_Hb | 14917844 | 14579986 | 2.2G | 2.2G | 0.02 | 98.24 | 94.91 | 53.42 |

**Additional file 2 RNA-Seq quality.** The Q20, Q30, GC-content, and sequence duplication level data.
