# Supplementary material for: Salmonella adhesion is decreased by hypoxia due to adhesion and motility structure crosstalk
Source: Vet Res. 2023 Oct 24;54:99. doi: 10.1186/s13567-023-01233-2 (PMC10598919; doi:10.1186/s13567-023-01233-2)
Supplement: Supplementary file 3 — Additional file 3. Salmonella strains characterization – growth curves. A. Growth curves in stationary growth conditions (without agitation) in normoxia and hypoxia (1% of oxygen) were obtained for all the generated mutants. B. Agglutination assay. Detection of T1F expression by microplate yeast agglutination test. The numbers indicate the geometric average of the titer endpoint (N = 5) for both Salmonella strains. [file 13567_2023_1233_MOESM3_ESM.docx]

**Additional file 3:
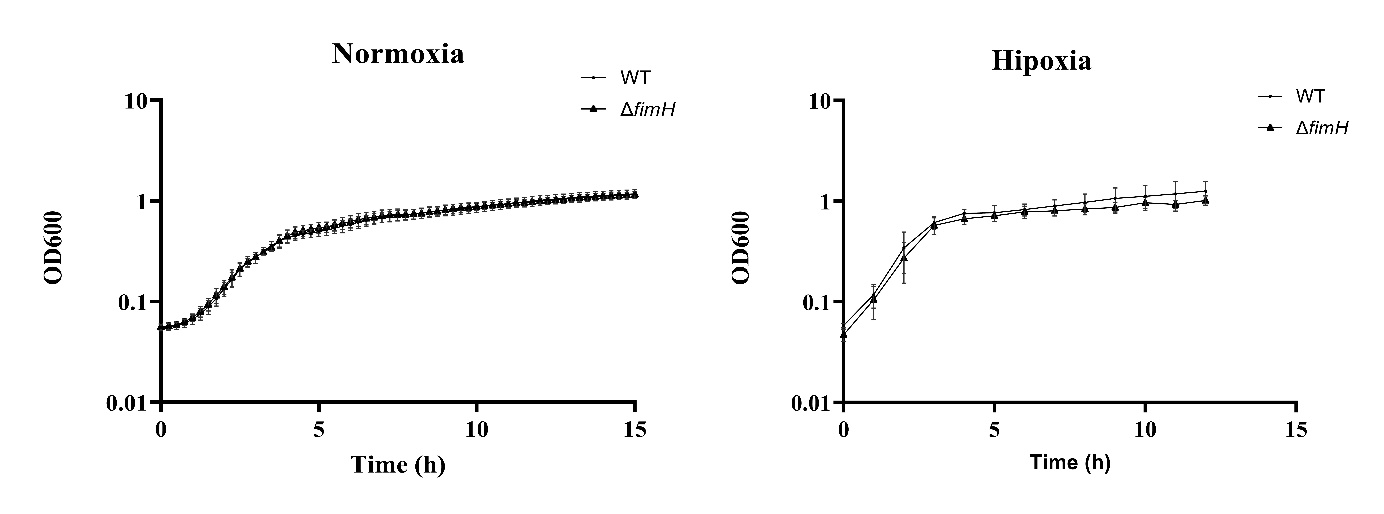
** ***Salmonella* strains characterization – growth curves**

**A Normoxia Hypoxia**

Additional file 3A. Growth curves in stationary growth conditions (without agitation) in normoxia and hypoxia (1% of oxygen) were obtained for all the generated mutants. Three independent experiments were made and error bars are indicated on the graph.

**B**


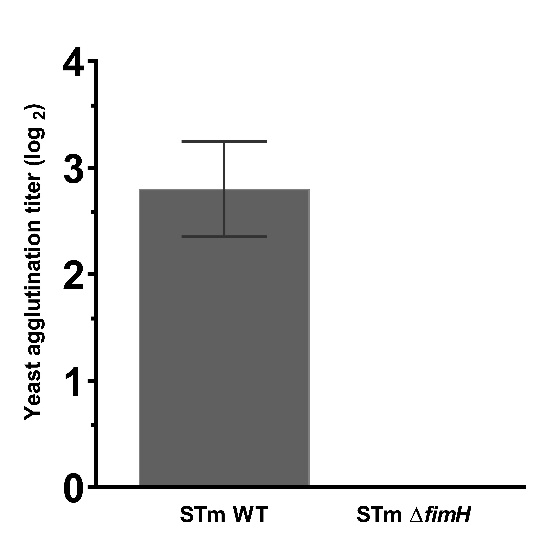


Additional file 3B. Agglutination assay. Detection of T1F expression by microplate yeast agglutination test. The numbers indicate the geometric average of the titer endpoint (*N* = 5) for both *Salmonella* strains.
