# Supplementary material for: Salmonella adhesion is decreased by hypoxia due to adhesion and motility structure crosstalk
Source: Vet Res. 2023 Oct 24;54:99. doi: 10.1186/s13567-023-01233-2 (PMC10598919; doi:10.1186/s13567-023-01233-2)
Supplement: Supplementary file 4 — Additional file 4. Hif-1α expression. A. Expression of Hif-1α mRNA in IPEC-J2 cell line following 2 h infection with MOI 100 of Salmonella Typhimurium wild type (STmWT) and T1F STmΔfimH) after the third passage in normal oxygen level (dark gray bars) and low oxygen level (light gray bars). Non-infected IPEC-J2 cells were assigned as a calibrator sample, and fold change was measured over unstimulated cells set at 1. Data represent the mean ± SD of at least three independent experiments. Triplicate samples were analyzed in each experiment to confirm the accuracy and reproducibility of qPCR. Statistical differences between strains were analyzed by Student t-test, and presented as individual values with a mean; there were non-significant differences between the samples. B. Western blot of IPEC-J2 cells grown in hypoxia, normoxia or in the presence of CoCL2. The presence of HIF-1α was assessed by Western blot analysis in IPEC-J2 cell lysates obtained by scraping the cells using SDS-PAGE buffer and sonication at 4 ℃ after 6 h incubation with CoCl2 and after 6 h of growth under normoxic and hypoxic conditions. As a reference beta-Actin Ab (Cell Signaling) was used. [file 13567_2023_1233_MOESM4_ESM.docx]

**Additional file 4: Hif-1α expression**


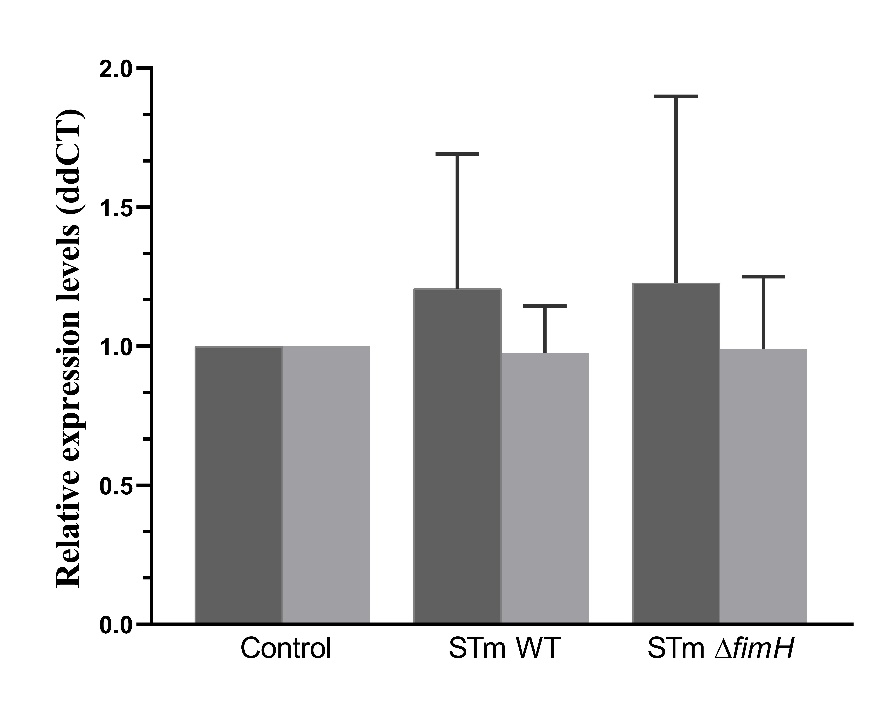
**A**

Additional file 4A. Expression of Hif-1α mRNA in IPEC-J2 cell line following 2 h infection with MOI 100 of *S.* Typhimurium wild type (STm WT) and T1F mutants (fimH^Pro57Leu^, fimH^Thr78Ile^, ΔfimH) after the third passage in normal oxygen level (dark gray bars) and low oxygen level (light gray bars). Non-infected IPEC-J2 cells were assigned as a calibrator sample, and fold change was measured over unstimulated cells set at 1. Data represent the mean ± SD of at least three independent experiments. Triplicate samples were analyzed in each experiment to confirm the accuracy and reproducibility of qPCR. Statistical differences between strains were analyzed by *t*-Student test, and presented as individual values with a mean; there were non-significant differences between the samples.

**B
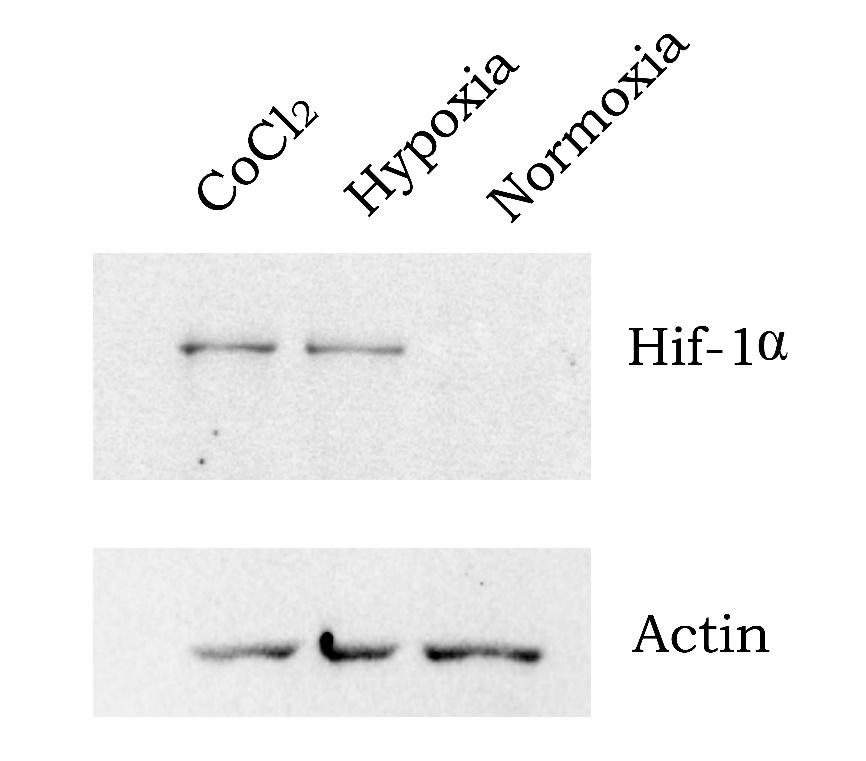
**

Additional file 4B. Western blot of IPEC-J2 cells grown in hypoxia, normoxia or in the presence of CoCL_2._ The presence of HIF-1α was assessed by Western blot analysis in IPEC-J2 cell lysates obtained by scraping the cells using SDS-PAGE buffer and sonication at 4 ℃ after 6 h incubation with CoCl_2_ and after 6 h of growth under normoxic and hypoxic conditions. As a reference beta-Actin Ab (Cell Signaling) was used.
