# Supplementary material for: Salmonella adhesion is decreased by hypoxia due to adhesion and motility structure crosstalk
Source: Vet Res. 2023 Oct 24;54:99. doi: 10.1186/s13567-023-01233-2 (PMC10598919; doi:10.1186/s13567-023-01233-2)
Supplement: Supplementary file 5 — Additional file 5. Salmonella Typhimurium 2 h incubation without cells. Salmonella. Typhimurium wild type (STmWT) and T1F mutants STmΔfimH (after the third passage were incubated for two hours in an infection medium in 24-well plates in normal oxygen level (dark gray bars) and low oxygen level (light gray bars). [file 13567_2023_1233_MOESM5_ESM.docx]

**Additional file 5:
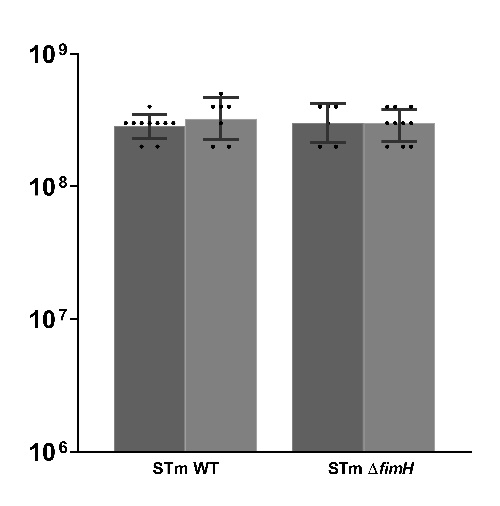
 *Salmonella* Typhimurium incubation in hypoxia**

**
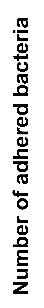
**

*Salmonella* Typhimurium 2 h incubation without cells. *S.* Typhimurium wild type (*S*TmWT) and T1F mutants *S*Tm*ΔfimH* (after the third passage were incubated for two hours in an infection medium in 24-well plates in normal oxygen level (dark gray bars) and low oxygen level (light gray bars).
