# Supplementary material for: Salmonella adhesion is decreased by hypoxia due to adhesion and motility structure crosstalk
Source: Vet Res. 2023 Oct 24;54:99. doi: 10.1186/s13567-023-01233-2 (PMC10598919; doi:10.1186/s13567-023-01233-2)
Supplement: Supplementary file 6 — Additional file 6. DEG (Differentially expressed genes). Comparison of differentially expressed genes involved in adhesion, invasion, and motility of Salmonella Typhimurium WT and ΔfimH mutant. [file 13567_2023_1233_MOESM6_ESM.docx]

**Additional file 6 DEGs (Differentially expressed genes)**

| *S*TmWT Normoxia vs *S*TmWT Hypoxia | | |
| --- | --- | --- |
| Gene name | **log_2_ fold change** | **Gene description** |
| bcfA | -1.17854736136676 | fimbrial subunit && PF00419:Fimbrial protein |
| fljB | 4.4070326004433 | flagellin && PF08884:Flagellin D3 domain\|PF00700:Bacterial flagellin C-terminal helical region\|PF00669:Bacterial flagellin N-terminal helical region |
| invA | 1.04589339122293 | secretory apparatus of type III secretion system && PF00771:FHIPEP family |
| invG | 1.26644272894916 | type III secretion system secretory apparatus && PF00263:Bacterial type II and III secretion system protein\|PF03958:Bacterial type II/III secretion system short domain |
| invJ | 1.10712336929219 | surface presentation of antigens protein (associated with type III secretion and virulence) && PF02510:Surface presentation of antigens protein |
| lpfA | -1.62495927528289 | long polar fimbria protein A (LpfA) && PF00419:Fimbrial protein |
| pefA | 1.93059566131925 | plasmid-encoded major fimbrial subunit && - |
| pefI | 1.04564836135167 | plasmid-encoded fimbriae && PF04703:FaeA-like protein |
| pilI | -3.32130383217808 | pilus protein && PF10623:Plasmid conjugative transfer protein PilI |
| pilJ | -3.16916661642671 | pilus protein && - |
| pilK | -3.47060342706479 | pilus protein && - |
| pilL | -4.09800402383419 | lipoprotein && PF10671:Toxin co-regulated pilus biosynthesis protein Q |
| pilM | -4.61399803331397 | pilus protein && PF07419:PilM |
| pilO | -3.68825383063639 | pilus protein && PF06864:Pilin accessory protein (PilO) |
| pilP | -4.23081985864808 | pilus protein && PF11356:Type IV pilus biogenesis |
| pilQ | -4.13604848081753 | pilus ATP-binding protein && PF00437:Type II/IV secretion system protein |
| pilR | -4.19583370987206 | pilus integral membrane protein && PF00482:Type II secretion system (T2SS) |
| pilS | -4.31322274195158 | typeIV prepilin && PF08805:PilS N terminal |
| pilT | -4.11248252774536 | pilus protein && PF01464:Transglycosylase SLT domain |
| pilV | -3.41739814649748 | typeIV prepilin && PF04917:Bacterial shufflon protein |
| pipC | 1.307360038573 | cell invasion protein && PF07824:Type III secretion chaperone domain |
| rtsA | 1.19192132425609 | Regulator of SPI-1 && PF12833:Helix-turn-helix domain |
| rtsB | 1.12500171431624 | Regulator of flhDC && PF00196:Bacterial regulatory proteins |
| sifB | 1.10511018686672 | Type III secretion system effector protein && PF06767:Sif protein |
| sipA | 1.28326647554634 | pathogenicity island 1 Type III secretion system effector protein-involved in actin bundling and polymerisation leading to epithelial cell invasion and formation of the SCV && PF09052:Salmonella invasion protein A |
| sipB | 1.18874602165437 | pathogenicity island 1 Type III secretion system effector protein && PF16535:Type III cell invasion protein SipB\|PF04888:Secretion system effector C (SseC) like family |
| sipC | 1.13082307126113 | pathogenicity island 1 Type III secretion system effector protein-involved in bacterial entry by actin bundling and part of the Translocon && PF09599:Salmonella-Shigella invasin protein C (IpaC_SipC) |
| sipD | 1.30781731278234 | pathogenicity island 1 Type III secretion system apparatus-part of the Translocon && PF06511:Invasion plasmid antigen IpaD |
| sopB | 1.20456617888846 | Type III secretion system effector protein. Activates Cdc42%2C RhoG%2C AktA and chloride secretion through its inositol phosphatase activity and disrupts tight junctions && PF05925:Enterobacterial virulence protein IpgD |
| sopE | 1.01086448299734 | Type III secretion system effector protein%2C invasion-associated secreted protein-by rearranging the actin cytoskeleton and disrupting tight junctions && PF05364:Salmonella type III secretion SopE effector N-terminus\|PF07487:SopE GEF domain |
| spaQ | 1.04576014141095 | type III secretion system secretory apparatus && PF01313:Bacterial export proteins |
| sptP | 1.04012174788602 | Type III secretion system effector protein%2C Inhibits Cdc42 and Rac1 by its GAP activity and MAPK signalling and IL-8 secretion through its tyrosine phosphatase activity && PF03545:Yersinia virulence determinant (YopE)\|PF00102:Protein-tyrosine phosphatase\|PF09119:SicP binding |
| ssaB | 1.40627397157229 | Type III secretion system effector protein%2C Interferes with endosomal trafficking && - |
| ssaO | -1.19046347778493 | Type III secretion system apparatus && - |
| sseL | 1.1437046082915 | Type III secretion system effector protein%2C deubiquitinase && - |
| stfA | -1.401360243419 | major fimbrial subunit stfa (hypothetical fimbrial subunit) && PF00419:Fimbrial protein |
| stiC | -1.17428400380238 | fimbrial usher protein && PF00577:Outer membrane usher protein\|PF13954:PapC N-terminal domain\|PF13953:PapC C-terminal domain |
| ybaY | -1.10681621305689 | conserved hypothetical lipoprotein && PF09619:Type III secretion system lipoprotein chaperone (YscW) |

| *S*Tm*ΔfimH* Normoxia *vs S*Tm*ΔfimH Hypoxia* | | |
| --- | --- | --- |
| Gene name | **log_2_ fold change** | **Gene description** |
| comE | 1.11804257810495 | competence gene-DNA binding and transport && PF00263:Bacterial type II and III secretion system protein\|PF03958:Bacterial type II/III secretion system short domain |
| fimA | 2.25379321932429 | type-1 fimbrial protein%2C a chain precursor && PF00419:Fimbrial protein |
| fimC | 1.89221113738416 | fimbrial chaperone protein && PF00345:Pili and flagellar-assembly chaperone, PapD N-terminal domain\|PF02753:Pili assembly chaperone PapD, C-terminal domain |
| fimD | 1.91986065166714 | outer membrane usher protein FimD precursor && PF13953:PapC C-terminal domain\|PF00577:Outer membrane usher protein\|PF13954:PapC N-terminal domain |
| fimF | 2.27148235690358 | hypothetical fimbrial protein in fimZ 5' region && PF00419:Fimbrial protein |
| fimH | 2.78984857329332 | FimH protein precursor && PF00419:Fimbrial protein |
| fimI | 2.14077112903124 | major pilin protein && PF00419:Fimbrial protein |
| fimW | 3.75324164194158 | fimbriae w protein && - |
| fimY | 1.64275457764796 | fimbriae Y protein && - |
| fimZ | 2.5374783461841 | probable transcriptional regulator (FimXZ protein) && PF00072:Response regulator receiver domain\|PF00196:Bacterial regulatory proteins, luxR family |
| flgA | -2.124021547228 | flagellar basal body P-ring protein FlgA precursor && PF13144:Chaperone for flagella basal body P-ring formation |
| flgB | -2.6588780155248 | hypothetical flagellar basal-body rod protein FlgB (proximal rod protein) && PF00460:Flagella basal body rod protein |
| flgC | -2.39328176689841 | hypothetical flagellar basal-body rod protein FlgC (proximal rod protein) && PF00460:Flagella basal body rod protein\|PF06429:Flagellar basal body rod FlgEFG protein C-terminal |
| flgD | -2.01088546570956 | flagellar hook formation protein FlgD && PF13861:FlgD Tudor-like domain\|PF03963:Flagellar hook capping protein - N-terminal region\|PF13860:FlgD Ig-like domain |
| flgE | -2.05995974077354 | flagellar hook protein FlgE && PF07559:Flagellar basal body protein FlaE\|PF06429:Flagellar basal body rod FlgEFG protein C-terminal\|PF00460:Flagella basal body rod protein |
| flgF | -2.24034505586755 | hypothetical flagellar basal-body rod protein FlgF (proximal rod protein) && PF06429:Flagellar basal body rod FlgEFG protein C-terminal\|PF00460:Flagella basal body rod protein |
| flgG | -2.32037083229116 | flagellar basal-body rod protein FlgG (distal rod protein) && PF00460:Flagella basal body rod protein\|PF06429:Flagellar basal body rod FlgEFG protein C-terminal |
| flgH | -2.37278580067362 | Flagellar L-ring protein precursor && PF02107:Flagellar L-ring protein |
| flgI | -2.58952740550724 | Flagellar P-ring protein precursor && PF02119:Flagellar P-ring protein |
| flgJ | -2.35026257108829 | flagellar protein FlgJ && PF10135:Rod binding protein\|PF01832:Mannosyl-glycoprotein endo-beta-N-acetylglucosaminidase |
| flhAa | -2.21178127168951 | flagellar biosynthesis protein FlhA && PF00771:FHIPEP family |
| flhB | -2.57483600838348 | flagellar biosynthetic protein FlhB && PF01312:FlhB HrpN YscU SpaS Family |
| flhE | -1.19242097903392 | flagellar protein FlhE precursor && PF06366:Flagellar protein FlhE |
| fliA | -1.86698036962066 | RNA polymerase sigma transcription factor for flagellar operon && PF04539:Sigma-70 region 3\|PF04545:Sigma-70, region 4\|PF04542:Sigma-70 region 2 |
| fliE | -2.73419122661636 | flagellar hook-basal body complex protein FliE && PF02049:Flagellar hook-basal body complex protein FliE |
| fliF | -2.60467009715286 | flagellar basal-body M-ring protein && PF08345:Flagellar M-ring protein C-terminal\|PF01514:Secretory protein of YscJ/FliF family |
| fliG | -2.29118255778693 | flagellar motor switch protein FliG && PF14841:FliG middle domain\|PF14842:FliG N-terminal domain\|PF01706:FliG C-terminal domain |
| fliH | -2.51819278806805 | flagellar assembly protein FliH && PF02108:Flagellar assembly protein FliH |
| fliI | -2.63727829078591 | flagellum-specific ATP synthase && PF00006:ATP synthase alpha/beta family, nucleotide-binding domain |
| fliJ | -2.37373156446238 | flagellar biosynthesis protein && PF02050:Flagellar FliJ protein |
| fliK | -2.14467581312329 | flagellar hook-length control protein && PF02120:Flagellar hook-length control protein FliK |
| fliL | -2.84380460415462 | flagella biosynthesis protein && PF03748:Flagellar basal body-associated protein FliL |
| fliM | -2.56214566151536 | flagellar motor switch protein FliM && PF02154:Flagellar motor switch protein FliM\|PF01052:Type III flagellar switch regulator (C-ring) FliN C-term |
| fliN | -2.73824572460904 | flagellar motor switch protein FliN && PF16973:Flagellar motor switch protein FliN N-terminal\|PF01052:Type III flagellar switch regulator (C-ring) FliN C-term |
| fliO | -2.68486023476284 | flagellar biosynthesis protein && PF04347:Flagellar biosynthesis protein, FliO |
| fliP | -2.73595199519028 | flagellar biosynthetic protein FliP && PF00813:FliP family |
| fliQ | -1.88453778671889 | flagellar biosynthetic protein FliQ && PF01313:Bacterial export proteins, family 3 |
| fliR | -1.84381414035289 | flagellar biosynthetic protein FliR && PF01311:Bacterial export proteins, family 1 |
| fliS | -2.12823273375929 | flagellar protein FliS && PF02561:Flagellar protein FliS |
| fliT | -2.12790026921281 | flagellar protein FliT && PF05400:Flagellar protein FliT |
| fliZ | -2.06772361873496 | FliZ protein && PF02899:Phage integrase, N-terminal SAM-like domain |
| fljA | -5.698640776609 | repressor of phase 1 flagellin gene && PF03614:Repressor of phase-1 flagellin |
| fljB | -7.20719905306083 | flagellin && PF08884:Flagellin D3 domain\|PF00700:Bacterial flagellin C-terminal helical region\|PF00669:Bacterial flagellin N-terminal helical region |
| invB | 1.0228298366882 | chaperone protein for type III secretion system effectors && PF03519:Invasion protein B family |
| invF | 1.11886553251646 | AraC-family regulatory protein && PF12833:Helix-turn-helix domain |
| motA | -1.30584452345771 | motility protein A && PF01618:MotA/TolQ/ExbB proton channel family |
| motB | -1.76231427070497 | motility protein B && PF00691:OmpA family\|PF13677:Membrane MotB of proton-channel complex MotA/MotB |
| pilB | -1.26478546264246 | competence gene-DNA binding and transport && PF00437:Type II/IV secretion system protein |
| pilJ | -1.50950219339341 | pilus protein && - |
| pilM | -1.02079980500645 | pilus protein && PF07419:PilM |
| pipC | 1.23219061530904 | cell invasion protein && PF07824:Type III secretion chaperone domain |
| sicA | 1.02522672744643 | type III secretion-associated chaperone && PF07720:Tetratricopeptide repeat |
| SL1344_2881 | 1.17708109276651 | salmonella pathogenicity island 1 protein && PF07377:Protein of unknown function (DUF1493) |
| sopB | 1.27788392304226 | Type III secretion system effector protein. Activates Cdc42%2C RhoG%2C AktA and chloride secretion through its inositol phosphatase activity and disrupts tight junctions && PF05925:Enterobacterial virulence protein IpgD |
| sopE | 1.18689046774617 | Type III secretion system effector protein%2C invasion-associated secreted protein-by rearranging the actin cytoskeleton and disrupting tight junctions && PF05364:Salmonella type III secretion SopE effector N-terminus\|PF07487:SopE GEF domain |
| sopE2 | 1.21547290175191 | Type III secretion system effector protein-causes membrane ruffling and disrupts tight junctions && PF07487:SopE GEF domain\|PF05364:Salmonella type III secretion SopE effector N-terminus |
| sseG | 1.35962638593995 | Type III secretion system effector protein-modulates the positioning of the SCV && - |
| sseL | 1.04769804047918 | Type III secretion system effector protein%2C deubiquitinase && - |
| stbB | 5.83708594031476 | fimbrial chaperone protein && PF00345:Pili and flagellar-assembly chaperone, PapD N-terminal domain\|PF02753:Pili assembly chaperone PapD, C-terminal domain |
| stdA | 3.92614157159959 | probable fimbrial protein && PF00419:Fimbrial protein |
| stdB | 1.96455759232877 | probable outer membrane fimbrial usher protein && PF13954:PapC N-terminal domain\|PF00577:Outer membrane usher protein\|PF13953:PapC C-terminal domain |
| stdC | 3.97213001612243 | probable fimbrial chaperone protein && PF02753:Pili assembly chaperone PapD, C-terminal domain\|PF00345:Pili and flagellar-assembly chaperone, PapD N-terminal domain |
| steB | 1.02223709155271 | Type III secretion system effector protein && - |
| stjC | 1.66913330724699 | fimbrial chaperone && PF02753:Pili assembly chaperone PapD, C-terminal domain\|PF00345:Pili and flagellar-assembly chaperone, PapD N-terminal domain |
| STM3026 | 1.31835148450077 | probable fimbrial membrane protein && - |
| STnc490 | 1.25235334673797 | small RNA%3B experimentally verified && PF10431:C-terminal, D2-small domain, of ClpB protein\|PF00004:ATPase family associated with various cellular activities (AAA)\|PF02861:Clp amino terminal domain, pathogenicity island component\|PF07724:AAA domain (Cdc48 subfamily) |
| ycgR | -1.60951344746878 | conserved hypothetical protein && PF07317:Flagellar regulator YcgR\|PF07238:PilZ domain |
