# Supplementary material for: Salmonella adhesion is decreased by hypoxia due to adhesion and motility structure crosstalk
Source: Vet Res. 2023 Oct 24;54:99. doi: 10.1186/s13567-023-01233-2 (PMC10598919; doi:10.1186/s13567-023-01233-2)
Supplement: Supplementary file 7 — Additional file 7. Stability of the expression of 16S reference. Stability of the expression of 16S reference gene in analyzed STm strains (WT and ΔfimH mutant) in both growth conditions (normoxia and hypoxia). The average Ct values within all biological replicates were around 10 and fluctuated less than one cycle (10.44 ± 0.74, 10.40 ± 0.57, 10.58 ± 0.6, 10.21 ± 0.73, respectively). [file 13567_2023_1233_MOESM7_ESM.docx]

**Additional file 7 Stability of the expression of 16S reference**


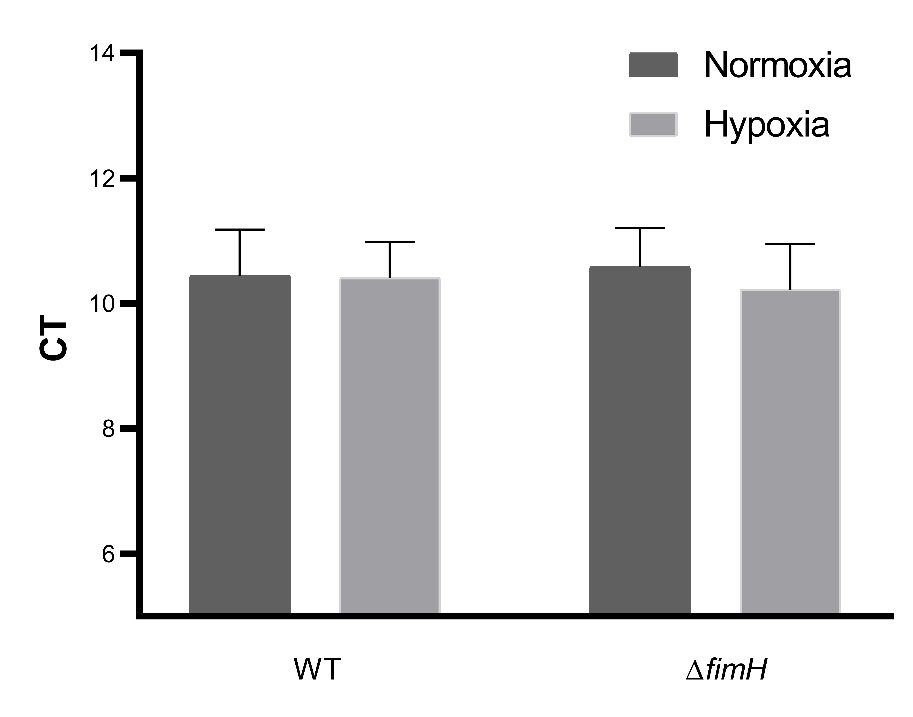


Stability of the expression of 16S reference gene in analyzed STm strains (WT and dfimH mutant) in both growth conditions (normoxia and hypoxia). The average Ct values within all biological replicates were around 10 and fluctuated less than one cycle (10.44 ± 0.74, 10.40 ± 0.57, 10.58 ± 0.6, 10.21 ± 0.73, respectively).
